# Polymorphisms in genes involved in the estrogen pathway and mammographic density

**DOI:** 10.1186/1471-2407-10-636

**Published:** 2010-11-22

**Authors:** Isabelle Dumas, Caroline Diorio

**Affiliations:** 1 Université Laval, Département de médecine sociale et préventive, Quebec City, QC, Canada; 2 Santé des populations: URESP, Centre de recherche FRSQ du CHA universitaire de Québec, Quebec City, QC, Canada; 3 Centre des maladies du sein Deschênes-Fabia, Hôpital du Saint-Sacrement, Quebec City, QC, Canada

## Abstract

**Background:**

Single nucleotide polymorphisms (SNPs) in genes involved in the estrogen pathway appear to be associated with breast cancer risk and possibly with mammographic density (MD), but little is known of these associations among premenopausal women. This study examines the association of 11 polymorphisms in five estrogen-related genes (estrogen receptors alpha and beta (*ERα, ERβ*), 17β-hydroxysteroid dehydrogenase 1 (*HSD17B1*), catechol-O-methyltransferase (*COMT*), cytochrome P450 1B1 (*CYP1B1*)) with premenopausal MD. Effect modification of four estrogen-related factors (parity, age at menarche, hormonal derivatives use and body mass index (BMI)) on this relation is also assessed.

**Methods:**

Polymorphisms were genotyped in 741 premenopausal Caucasian women whose MD was measured in absolute density (AD, cm^2^) and percent density using a computer-assisted method. Multivariate linear models were used to examine the associations (*P*_trend_) and interactions (*P*_i_).

**Results:**

None of the SNPs showed a statistically significant association with AD. However, each additional rare allele of rs1056836 *CYP1B1 *was associated with a reduction in AD among nulliparous women (*P*_trend _= 0.004), while no association was observed among parous women (*P*_trend _= 0.62; *P*_i _= 0.02). An increase in the number of rare alleles of the *HSD17B1 *SNP (rs598126 and rs2010750) was associated with an increase in AD among women who never used hormonal derivatives (*P*_trend _= 0.06 and *P*_trend _= 0.04, respectively), but with a decrease in AD among past hormonal derivatives users (*P*_trend _= 0.04; *P*_i _= 0.02 and *P*_trend _= 0.08; *P*_i _= 0.01, respectively). Moreover, a negative association of rs598126 *HSD17B1 *SNP with AD was observed among women with higher BMI (>median) (*P*_trend _= 0.01; *P*_i _= 0.02). A negative association between an increased number of rare alleles of *COMT *rs4680 SNP and AD was limited to women who never used hormonal derivatives (*P*_trend _= 0.02; *P*_i _= 0.03) or with late age at menarche (>median) (*P*_trend _= 0.03; *P*_i _= 0.02). No significant association was observed between polymorphisms in the *ERα *or *ERβ *genes and AD. Similar results, although less significant, were observed when MD was assessed in percent density.

**Conclusion:**

SNPs located in *CYP1B1*, *COMT *or *HSD17B1 *genes seem to be associated with MD in some strata of estrogen-related factors. Our findings suggest that modifying effects of estrogen-related factors should be considered when evaluating associations of polymorphisms in estrogen-related genes with premenopausal mammographic density.

## Background

There is substantial evidence that steroid hormones such as estrogens play an important role in the etiology of breast cancer. In particular, estrogens are believed to contribute to tumor growth by promoting the proliferation of cells with existing mutations or perhaps by increasing the opportunity for mutations [1]. Therefore, several enzymes and receptors involved in the estrogen pathway have been suggested to play a role in the development of breast cancer [1,2]. Among them, 17β-hydroxysteroid dehydrogenase 1 (HSD17B1) is the enzyme responsible for the conversion of estrone to estradiol [3] which is the most potent estrogen. Cytochrome P450 1B1 (CYP1B1) catalyses the conversion of estrone and estradiol to potentially carcinogenic catechol estrogen 4-hydroxyestrogen (4-OH) [4-6]. The catechol-O-methyltransferase (COMT) enzyme is principally responsible for both the inactivation and detoxification of catecholestrogens, which can cause oxidative damage [4,7]. Both estrogen receptors alpha and beta (ERα, ERβ) are members of the nuclear receptor family of ligand-inducible transcription factors, but they are believed to have different transcriptional activation properties [8]. The interaction of estrogen and estrogen receptors stimulates cell proliferation which is believed to be one of the mechanisms responsible for the carcinogenicity of estrogens in the human breast [1]. Specific polymorphisms (SNPs) in these estrogen-related genes could directly or indirectly lead to variations in their activities and therefore, may have an effect on breast cancer risk. For instance, certain SNPs located in *ERα*, *ERβ*, *HSD17B1*, *COMT *and *CYP1B1 *genes have been associated with breast cancer risk among premenopausal or postmenopausal women [9-12] or in some strata of other estrogen-related factors such as parity, hormone replacement therapy (HRT) use, age at menarche or body mass index (BMI) [9,13-15].

Those SNPs located in *ERα*, *ERβ*, *HSD17B1*, *COMT *and *CYP1B1 *genes may also be associated to mammographic density (MD), which is a strong and independent risk factor for breast cancer [16]. It has been repeatedly found that women with 75% or more of MD have a four to six fold greater risk of developing breast cancer compared to women with no measurable dense breast tissue [16,17]. Elevated MD represents a higher proportion of fibroglandular cells and therefore reflects a higher proliferative activity within this tissue [18]. The link between MD and breast cancer is not well understood but it could be related to estrogen. For instance, in addition to the proliferative effect on fibroglandular cells of the breast by endogenous or exogenous estrogens [1,19], several breast cancer risk factors related to estrogens such as age, parity, menopausal status or HRT use, are also associated with MD [16,17]. Because MD is a highly heritable factor [20], a number of studies examined the association of SNPs located in estrogen-related genes with MD [21-33]. In these studies, although no associations were found among the entire population, some associations were observed in certain strata of estrogen-related factors such as menopausal status or the use of HRT [23,28,29,31,32]. Up to now, few studies were conducted among premenopausal women [28-33] and no data is available for stratified analysis based on estrogen-related factors, even though heritability of MD has been suggested to be higher in this population [34]. The purpose of this study was to evaluate the relation between 11 polymorphisms in five genes involved in the estrogen pathway (*ERα*, *ERβ*, *HSD17B1*, *COMT *and *CYP1B1*) and premenopausal MD, and to examine the modifying effect of four estrogen-related factors (parity, hormonal derivatives use, age at menarche and BMI) on these associations.

## Methods

### Study Population and Recruitment Procedures

Details of the study design and methods have been published elsewhere [35]. In summary, subjects participating in the present study were premenopausal women who received a screening mammography between February and December 2001 at the Clinique Radiologique Audet (Quebec City, QC, Canada). Based on previously published criteria [36], to be qualified as premenopausal, women had to have had at least one menstrual cycle within previous twelve months, or they had to be younger than 48 years old for a nonsmoker or 46 years old for a smoker after hysterectomy without bilateral oophorectomy. Other restrictions were that women had to have not taken hormonal derivatives within 3 months prior to mammography, never have used tamoxifen or raloxifene, not be pregnant, have no history of cancer at any site, have had no breast surgery and no endocrine system disease. Among the 783 premenopausal eligible women, 37 women did not give authorization for blood banking of samples for further study. Therefore a total of 746 women were included in the present study. The research ethics committee of the CH*A *universitaire de Québec (Quebec City, QC, Canada) reviewed and approved this study. A written informed consent was provided by all study participants.

### Data Collection

Anthropometric measurements and blood samples were taken at recruitment. Breast cancer risk factors including reproductive and menstrual history, family history of breast cancer, personal history of breast biopsies, past use of hormonal derivatives, smoking status, alcohol intake, physical activity and education, were documented by a telephone interview. Diet was assessed with a self-administered semiquantitative food-frequency questionnaire [37].

### Digitization of Mammograms and Assessment of Mammographic Breast Density

A craniocaudal view of a randomly chosen breast was evaluated for each women after all mammograms were digitized using a Kodak Lumiscan85 digitizer at 260 μm per pixel. The proportion of the breast showing tissue density (percent density, PD in%) and the absolute amount of dense tissue (absolute density, AD in cm²) were assessed by one trained author (C.D.), with a computer-assisted method [38]. The evaluator did not have any information regarding women's status or medical history. The within-batch intraclass correlation coefficients (n = 210 duplicate images) were 0.98 and 0.98 for percent and absolute density, respectively. The between-batch coefficients of variation (n = 10 images repeated 21 times) were 4% for percent density and 5% for absolute density.

### DNA Extraction and SNP Genotyping

These procedures were described previously [39]. Briefly, DNA was extracted from the buffy coat using the PUREGene DNA extraction kit (Gentra Inc, Minneapolis, MN, USA) following the manufacturer's protocol. Genomic DNA of five women could not be obtained. DNA samples were blindly genotyped for 12 SNPs located in five genes involved in the estrogen pathway: *ERα*, *ERβ*, *HSD17B1*, *COMT *and *CYP1B1*. To be included in the present study, SNPs had to be located in promoter regions or exons, or found to be associated with breast cancer risk or its associated risk factors in previous studies [11,40,41]. From the 12 SNPs selected, 11 have been successfully genotyped and described in Table 1. The SNP not genotyped is rs605059 located in *HSD17B1 *gene. SNPs analyses were performed using the GenomeLab SNPstream (Beckman Coulter) or Sequenom MassArray (San Diego, Inc.) genotyping platforms according to the manufacturer's protocols. Each 96-well plate included negative (no DNA) and positive controls to ensure accuracy of genotyping. Genotyping call rates for both technologies ranged between 98.2% and 99.5%. In this study, all genotypes were repeated on 21 blind samples; concordance was 100% for both platforms. Protocols could be provided upon request from the corresponding author.

**Table 1:** Single nucleotide polymorphisms evaluated in the present study

Gene name	Encoded product	N	Alleles (major > minor)	**SNP reference ID **^**a**^	Gene Location	Panel	Call Rate
ERα	Estrogen receptor α	728	T > C	rs2077647	Exon 1	SNPstream	98.2%
		735	A > G	rs2234693	Intron 1	SNPstream	99.2%
		735	A > G	rs9340799	Intron 1	SNPstream	99.2%
		736	C > T	rs2228480	Exon 8	SNPstream	99.3%
ERβ	Estrogen receptor β	735	T > C	rs3829768	Promoter	SNPstream	99.2%
		737	G > A	rs1256049	Exon 6	SNPstream	99.5%
HSD17B1	17β-hydroxysteroid dehydrogenase 1	737	G > T	rs676387	Intron 4	Sequenom	99.5%
		732	C > T	rs598126	COASY : Exon	Sequenom	98.8%
		734	G > A	rs2010750	TCFL4 : Intron	Sequenom	99.1%
COMT	Catechol-O-methyltransferase	735	G > A	rs4680	Exon 4	Sequenom	99.2%
CYP1B1	Cytochrome P450 1B1	737	C > G	rs1056836	Exon 3	Sequenom	99.5%

^a ^Polymorphisms are identified by their dbSNP accession number at http://www.ncbi.nlm.nih.gov/SNP/.

### Statistical Methods

Deviation from the Hardy-Weinberg equilibrium was assessed for each SNP by a χ2 test with one degree of freedom, and linkage disequilibrium strength was evaluated with Lewontin's D' statistic for within-gene SNP pairs. In the present study, no significant deviation from Hardy-Weinberg expectations was observed for any of the polymorphisms (*P *> 0.05), and all of the D' values were > 0.88 except for pairs of rs2228480 SNP with others located in *ERα *(all D' < 0.19). Generalized linear models were used to estimate univariate and multivariate-adjusted least-square means of MD by category of genotypes under codominant mode of inheritance. Linear regression models were used to evaluate associations (*P*_trend_) between the number of copies of the rare allele entered as a continuous variable (0, 1, 2) and MD. Univariate and multivariate haplotype analyses were done based on SNPs in region of strong linkage disequilibrium within gene using the method described by Stram et al. [42]. For these analyses, the most common haplotype was used as the reference category, and rare haplotypes (frequency < 5%) were combined. All of the variables met the assumption of normality of residuals from these analysis with the exception of absolute density, which was square root transformed and presented as back-transformed values.

Since the activities of enzymes and receptors related to the studied genes are regulated by estrogens [43-45], the modifying effect of four estrogen-related factors on the associations between genotype and MD was assessed. These factors are parity (ever/never having a full-term pregnancy), hormonal derivatives used (ever/never having used oral contraceptive or hormone replacement therapy), age at menarche (≤ median/> median), and BMI (≤ median/> median), and they were selected because they were found as effect modifiers of some associations between SNPs in estrogen-related genes and breast cancer risk in previous studies [9,13-15]. An interaction term between each of these variables and the genotype dosage was added to the above models. The p-value of the interaction term (*P*_i_) was used to assess the effect modification of these variables individually. The modifying effect of these factors was also evaluated in haplotype analysis.

Models were adjusted for potential confounders including age at mammography (years), body mass index (kg/m^2^), waist-to-hip ratio, height (cm), age at menarche (years), age at first birth (years), number of full-term pregnancies, breastfeeding (months), number of breast biopsies, family history of breast cancer in first-degree relatives (yes or no), duration of past contraceptive and hormone replacement therapy uses (years), smoking status (never, former or current smoker), energy (Kcal/day) and alcohol (drinks/week) intakes, physical activity (metabolic equivalent hours/week) and education (completed elementary school, high school, college or university). Further adjustments were made (season at time of mammography, multivitamin-multimineral supplement use, phase of the menstrual cycle at time of mammography, dietary calcium and vitamin D intakes, plasma insulin-like growth factor-I and insulin-like growth factor-binding protein-3 levels) with little or no influence on the estimates. Therefore, they were not added in the models.

All statistical analyses were performed using the SAS software package (version 9.2; SAS Institute Inc.). All tests were 2-sided and a p-value < 0.05 was considered statistically significant.

## Results

Characteristics of the study population are summarized in Table 2. Of the 741 premenopausal women, 739 reported to be Caucasian (99.7%). The mean age at the time of mammogram was 46.8 years (standard deviation (SD) 4.6 years). The mean percent and absolute density were 42.3% (SD 24.3%) and 47.1 cm^2 ^(SD 28.7 cm^2^) respectively.

**Table 2:** Characteristics of study subjects (N = 741)

Characteristic	Mean	SD
Age at mammography (years)	46.8	4.6
Body mass index (kg/m^2^)	25.2	4.6
Waist-to-hip ratio	0.8	0.1
Height (cm)	160.5	5.8
Age at menarche (years)	12.7	1.6
Age at first pregnancy (years) ^a ^(n = 563)	26.0	3.7
Number of live births ^a ^(n = 563)	2.1	0.8
Alcohol intake (drinks/wk) (n = 738)	3.4	3.8
Total energy intake (Kcal/day) (n = 736)	1906.0	514.9
Physical activity (MET-h/wk) (n = 740)	27.1	22.2
Percent density (%)	42.3	24.3
Absolute density (cm²)	47.1	28.7
	%	

Parity (parous)	76.0	
Lactation (yes) ^a ^(n = 563)	62.0	
Personal history of breast biopsies (yes)	14.2	
Past hormonal derivative use (yes) (n = 740) ^b^	92.4	
First degree relative with breast cancer (yes)	36.8	
Education (college or university degree)	62.0	
Smoking status (never)	45.1	

^a ^Among parous women

^b ^Past oral contraceptive (92.2%) and hormone replacement therapy (6.1%) uses

Relation between polymorphisms in genes involved in the estrogen pathway and MD are presented in Table 3. Women carrying increased number of copies of the rare allele of *CYP1B1 *rs1056836 SNP had decreased adjusted means of percent (*P*_trend _= 0.09) and absolute (*P*_trend _= 0.08) density. However, no SNP in *ERα*, *ERβ*, *HSD17B1*, *COMT *and *CYP1B1 *genes was significantly associated to percent or absolute density (all *P*_trend _≥ 0.05). Moreover, we observed no significant association of any haplotypes within *ERα *or *HSD17B1 *genes with percent or absolute density (data not shown).

**Table 3:** Associations between SNPs in genes involved in the estrogen pathway and mammographic density

				Mean mammographic density			
				
				**Percent density (%) (95% CI)**^**a**^		**Absolute density (cm**^**2**^**)**^**b **^**(95% CI)**	
				
Gene name	**SNP reference ID**^**c**^	Genotype	**N (%)**^**d**^	Crude	**Adjusted**^**e**^	Crude	**Adjusted**^**e**^
ERα	rs2077647	TT	189 (25.96)	42.5 (39.0-46.0)	42.5 (39.7-45.4)	47.7 (43.6-51.9)	47.6 (43.8-51.7)
		TC	357 (49.04)	42.5 (40.0-45.1)	42.5 (40.5-44.6)	47.8 (44.8-50.8)	47.1 (44.3-50.0)
		CC	182 (25.00)	42.6 (39.0-46.1)	43.3 (40.4-46.3)	45.5 (41.5-49.7)	44.8 (40.9-48.8)
		*P*_trend_^f^		0.98	0.69	0.48	0.31
	rs2234693	AA	211 (28.71)	41.6 (38.3-44.8)	42.3 (39.5-45.0)	48.0 (44.1-52.0)	48.3 (44.6-52.2)
		AG	350 (47.62)	42.6 (40.1-45.2)	42.1 (40.0-44.2)	47.6 (44.7-50.7)	46.7 (43.9-49.7)
		GG	174 (23.67)	42.7 (39.1-46.3)	43.7 (40.7-46.7)	45.2 (41.1-49.4)	44.3 (40.4-48.4)
		*P*_trend_		0.63	0.50	0.35	0.16
	rs9340799	AA	301 (40.95)	42.1 (39.3-44.8)	42.7 (40.5-45.0)	49.1 (45.8-52.5)	49.4 (46.2-52.6)
		AG	327 (44.49)	42.2 (39.6-44.9)	41.8 (39.6-43.9)	45.8 (42.8-49.0)	44.6 (41.7-47.5)
		GG	107 (14.56)	43.9 (39.3-48.5)	44.9 (41.1-48.7)	46.6 (41.4-52.2)	46.2 (41.2-51.6)
		*P*_trend_		0.58	0.58	0.26	0.11
	rs2228480	CC	508 (69.02)	41.5 (39.4-43.6)	42.0 (40.3-43.8)	46.3 (43.9-48.9)	46.1 (43.8-48.5)
		CT	207 (28.13)	44.4 (41.1-47.7)	43.4 (40.7-46.1)	48.6 (44.7-52.6)	46.9 (43.2-50.8)
		TT	21 (2.85)	43.0 (32.6-53.4)	47.3 (38.7-55.9)	53.2 (40.9-67.2)	56.6 (44.3-70.5)
		*P*_trend_		0.20	0.20	0.19	0.25
ERβ	rs3829768	TT	733 (99.73)	42.4 (40.6-44.1)	42.6 (41.2-44.0)	47.2 (45.1-49.3)	46.7 (44.7-48.7)
		TC	2(0.27)	30.3 (-3.4-64.0)	16.5 (-11.2-44.2)	45.6 (15.9-94.2)	34.0 (10.6-74.7)
		*P*_trend_		0.48	0.07	0.94	0.48
	rs1256049	GG	688 (93.35)	42.6 (40.8-44.4)	42.8 (41.3-44.2)	47.4 (45.3-49.6)	46.9 (44.8-48.9)
		GA	48 (6.51)	38.9 (32.0-45.8)	40.0 (34.3-45.6)	44.2 (36.7-52.4)	44.5 (37.3-52.5)
		AA	1 (0.14)	43.0 (-4.7-90.7)		54.1 (12.1-133.8)	
		*P*_trend_		0.33	0.35	0.50	0.57
HSD17B1	rs676387	GG	390 (52.92)	42.2 (39.8-44.6)	41.7 (39.8-43.7)	46.2 (43.4-49.0)	45.1 (42.5-47.8)
		GT	288 (39.08)	42.8 (40.0-45.6)	43.4 (41.1-45.7)	48.4 (45.1-51.8)	48.4 (45.2-51.7)
		TT	59 (8.01)	40.1 (33.9-46.3)	42.6 (37.4-47.9)	46.4 (39.5-54.0)	47.3 (40.4-54.8)
		*P*_trend_		0.83	0.39	0.53	0.20
	rs598126	CC	195 (26.64)	41.2 (37.8-44.7)	42.6 (39.8-45.5)	48.2 (44.2-52.4)	48.4 (44.5-52.5)
		CT	354 (48.36)	43.7 (41.1-46.2)	43.1 (41.0-45.2)	47.6 (44.6-50.6)	46.6 (43.8-49.5)
		TT	183 (25.00)	40.5 (37.0-44.1)	41.0 (38.1-43.9)	44.5 (40.6-48.6)	44.3 (40.5-48.3)
		*P*_trend_		0.81	0.44	0.21	0.15
	rs2010750	GG	237 (32.29)	41.9 (38.8-45.0)	42.8 (40.3-45.4)	47.7 (44.1-51.5)	47.5 (44.0-51.1)
		GA	351 (47.82)	43.6 (41.0-46.1)	43.1 (41.0-45.1)	48.0 (45.0-51.1)	47.3 (44.5-50.2)
		AA	146 (19.89)	40.2 (36.3-44.2)	41.0 (37.7-44.2)	44.2 (39.9-48.8)	44.0 (39.7-48.4)
		*P*_trend_		0.67	0.46	0.30	0.26
COMT	rs4680	GG	190 (25.85)	43.1 (39.6-46.6)	42.4 (39.5-45.3)	48.0 (44.0-52.2)	47.7 (43.8-51.8)
		GA	375 (51.02)	43.3 (40.8-45.8)	43.8 (41.7-45.8)	47.1 (44.3-50.1)	46.5 (43.7-49.3)
		AA	170 (23.13)	39.0 (35.3-42.6)	39.6 (36.6-42.6)	45.7 (41.6-50.1)	45.4 (41.5-49.6)
		*P*_trend_		0.12	0.21	0.45	0.43
CYP1B1	rs1056836	CC	219 (29.72)	43.7 (40.4-46.9)	43.7 (41.0-46.3)	49.6 (45.8-53.6)	48.8 (45.2-52.6)
		CG	372 (50.47)	42.3 (39.8-44.8)	42.8 (40.8-44.8)	46.7 (43.8-49.6)	46.4 (43.7-49.2)
		GG	146 (19.81)	40.2 (36.2-44.1)	39.8 (36.6-43.1)	44.2 (39.9-48.9)	43.6 (39.4-48.0)
		*P*_trend_		0.18	0.09	0.07	0.08

^a ^CI: confidence interval. ^b ^Means of absolute density are presented as back-transformed values.

^c ^SNPs: single nucleotide polymorphisms. They are identified by their dbSNP accession number at http://www.ncbi.nlm.nih.gov/SNP/.

^d ^N (%) values are slightly lower in adjusted models because of ten missing values in adjustment variables. ^e ^Adjusted for age at mammography, body mass index, waist-to-hip ratio, height, age at menarche, age at first birth, number of full-term pregnancies, breastfeeding, number of breast biopsies, family history of breast cancer, past contraceptive and hormone replacement therapy uses, smoking status, energy and alcohol intakes, physical activity and education. ^f ^*P *value is the *P *trend, testing genotype dosage, number of copies of the rare allele entered as 0, 1, 2 and mammographic density entered as continuous variable.

Table 4 presents the effect modification of parity, hormonal derivatives used, age at menarche and BMI found statistically significant on the relation between polymorphisms in estrogen-related genes and MD. The results from these analyses regardless of statistical significance are detailed in Additional files 1, 2, 3 and 4. Among nulliparous women, an increase of rare alleles of *CYP1B1 *rs1056836 SNP was negatively associated with AD (*P*_trend _= 0.004), while no association was observed among parous women (*P*_trend _= 0.62; *P*_i _= 0.02). Effect modification of parity on the association between *COMT *rs4680 SNP and AD was also observed (*P*_i _< 0.05), but no association reached statistical significance among nulliparous (*P*_trend _= 0.15) or parous (*P*_trend _= 0.12) women. We found that an increase in the number of rare alleles of the *HSD17B1 *SNP (rs598126 and rs2010750) was associated with a decrease in AD among women who ever used hormonal derivatives (*P*_trend _= 0.04 and *P*_trend _= 0.08, respectively), but with an increase in AD among women who never used them (*P*_trend _= 0.06; *P*_i _= 0.02 and *P*_trend _= 0.04; *P*_i _= 0.01, respectively). Moreover, among women who never used hormonal derivatives, an increase in the number of rare alleles of the *COMT *rs4680 SNP was found to be negatively associated with AD (*P*_trend _= 0.02; *P*_i _= 0.03). These associations and effect modifications remain similar when the analysis is limited to women who ever or never used oral contraceptive (data not shown). Effect modification of age at menarche on the association of *COMT *rs4680 SNP with AD was also observed (*P*_i _= 0.02), revealing a negative association between an increase of rare alleles of this genotype and AD limited to women with an age at menarche above the median value (*P*_trend _= 0.03). Among women with a BMI above the median value, an increase in the number of rare alleles of the *HSD17B1 *rs598126 SNP was associated with a decrease in AD (*P*_trend _= 0.01), while no association was observed among those with a BMI below or equal to the median value (*P*_trend _= 0.53; *P*_i _= 0.02). When percent density was used as the dependent variable, similar trends were observed but they were less statistically significant. No significant effect modification of estrogen-related factors on the association of haplotypes within *ERα *or *HSD17B1 *genes with percent or absolute MD was observed (data not shown).

**Table 4:** Modifying effect of estrogen-related factors on the association between SNPs and mammographic density

SNP reference					Adjusted mean mammographic density (95% CI) ^a^			
					
Gene name	ID ^b^	Genotype	N (%)		Percent density (%)		Absolute density (cm^2^) ^c^	
**Parity**:			**No**	**Yes**	**No**	**Yes**	**No**	**Yes**

COMT	rs4680	GG	44 (6.1)	143 (19.7)	43.1 (37.1-49.1)	42.0 (38.7-45.3)	43.3 (35.7-51.7)	48.8 (44.3-53.6)
		GA	96 (13.2)	275 (38.0)	45.3 (41.3-49.3)	43.3 (41.0-45.7)	46.4 (41.1-52.1)	46.7 (43.5-50.1)
		AA	34 (4.7)	133 (18.3)	43.4 (36.7-50.0)	38.6 (35.2-42.0)	52.6 (43.3-62.9)	43.6 (39.3-48.2)
		*P*_trend _^d^			0.93	0.17	0.15	0.12
		*P*_i _^e^			0.47		0.05	
CYP1B1	rs1056836	CC	53 (7.3)	163 (22.4)	46.6 (41.1-52.1)	42.8 (39.7-45.8)	54.2 (46.4-62.7)	47.2 (43.1-51.5)
		GC	86 (11.8)	282 (38.8)	46.3 (42.0-50.5)	41.8 (39.5-44.1)	47.0 (41.3-53.0)	46.4 (43.3-49.6)
		GG	35 (4.8)	108 (14.8)	36.9 (30.3-43.5)	40.6 (36.8-44.3)	37.0 (29.5-45.4)	45.5 (40.6-50.7)
		*P*_trend_			0.04	0.38	0.004	0.62
		*P*_i_			0.19		0.02	

**Hormonal derivatives used**:			**No**	**Yes**	**No**	**Yes**	**No**	**Yes**

HSD17B1	rs598126	CC	14 (1.9)	177 (24.5)	38.3 (27.7-49.0)	43.0 (40.1-45.9)	38.1 (26.3-52.4)	49.3 (45.2-53.5)
		CT	24 (3.3)	326 (45.2)	44.2 (36.2-52.2)	43.0 (40.9-45.2)	45.4 (35.2-56.9)	46.6 (43.7-49.7)
		TT	16 (2.2)	165 (22.9)	52.0 (42.1-61.8)	39.9 (36.9-42.9)	56.6 (42.6-72.7)	43.1 (39.2-47.2)
		*P*_trend_			0.06	0.16	0.06	0.04
		*P*_i_			0.03		0.02	
HSD17B1	rs2010750	GG	15 (2.1)	218 (30.1)	38.2 (27.9-48.5)	43.1 (40.5-45.8)	37.9 (26.5-51.7)	48.2 (44.6-52.0)
		AG	25 (3.5)	322 (44.5)	44.7 (36.8-52.5)	42.9 (40.8-45.1)	45.8 (35.8-57.2)	47.4 (44.4-50.4)
		AA	15 (2.1)	129 (17.8)	52.9 (42.8-63.0)	39.6 (36.2-43.0)	58.7 (44.1-75.7)	42.4 (38.0-47.0)
		*P*_trend_			0.04	0.16	0.04	0.08
		*P*_i_			0.02		0.01	
COMT	rs4680	GG	15 (2.1)	172 (23.7)	47.5 (37.3-57.6)	41.8 (38.9-44.8)	55.0 (40.9-71.4)	47.0 (43.0-51.2)
		GA	29 (4.0)	342 (47.1)	49.1 (41.7-56.6)	43.3 (41.2-45.4)	50.2 (40.2-61.5)	46.1 (43.3-49.0)
		AA	11 (1.5)	156 (21.5)	31.8 (20.1-43.6)	40.2 (37.1-43.3)	30.6 (19.4-44.9)	46.6 (42.5-51.0)
		*P*_trend_			0.07	0.50	0.02	0.93
		*P*_i_			0.12		0.03	

**Age at menarche (median) (years)**:			**≤12**	**> 12**	**≤12**	**> 12**	**≤12**	**> 12**

COMT	rs4680	GG	76 (10.8)	108 (15.3)	41.2 (36.7-45.6)	43.7 (39.9-47.5)	42.7 (37.0-48.8)	51.6 (46.2-57.2)
		GA	165 (23.3)	196 (27.7)	43.0 (40.0-46.1)	43.9 (41.1-46.7)	48.7 (44.5-53.1)	43.7 (40.1-47.5)
		AA	73 (10.3)	89 (12.6)	40.8 (36.2-45.4)	38.2 (34.1-42.4)	47.9 (41.7-54.6)	43.4 (38.1-49.0)
		*P*_trend_			0.93	0.06	0.22	0.03
		*P*_i_			0.25		0.02	

**Body mass index (median) (kg/m**^**2**^**)**:			**≤24.4**	**> 24.4**	**≤24.4**	**> 24.4**	**≤24.4**	**> 24.4**

HSD17B1	rs598126	CC	91 (12.6)	100 (13.9)	49.8 (45.5-54.1)	34.5 (30.4-38.6)	49.1 (43.3-55.2)	47.1 (41.7-52.9)
		CT	188 (26.0)	162 (22.4)	51.3 (48.3-54.3)	34.7 (31.5-38.0)	50.0 (45.8-54.3)	43.7 (39.5-48.0)
		TT	92 (12.7)	89 (12.3)	52.3 (48.0-56.6)	29.1 (24.7-33.4)	51.9 (45.9-58.2)	37.2 (32.2-42.6)
		*P*_trend_			0.42	0.08	0.53	0.01
		*P*_i_			0.07		0.02	

^a ^Analyses are adjusted for age at mammography, body mass index, waist-to-hip ratio, height, age at menarche, number of breast biopsies, family history of breast cancer, past contraceptive and hormone replacement therapy uses, smoking status, energy and alcohol intakes, physical activity and education when applicable. Except for modifying effect of parity, models were also adjusted for age at first birth, number of full-term pregnancies and breastfeeding. CI: confidence interval.

^b ^SNPs: single nucleotide polymorphisms. They are identified by their dbSNP accession number at http://www.ncbi.nlm.nih.gov/SNP/.

^c ^Means of absolute density are presented as back-transformed values.

^d ^*P *value is the *P *trend, testing genotype dosage, number of copies of the rare allele entered as 0, 1, 2 and mammographic density entered as a continuous variable.

^e ^*P *value for interaction between the variable (parity, hormonal derivative used, age at menarche or body mass index) and the genotype dosage from linear regression.

## Discussion

In the present study, we evaluated the association of 11 polymorphisms in five genes involved in the estrogen pathway with MD, and found no overall association in premenopausal women. However, some SNPs located in the *CYP1B1*, *COMT *or *HSD17B1 *genes were found to be associated with MD among nulliparous women, non-hormonal users, those with late menarche or among women with high BMI. These results highlight the need to consider the modifying effect of estrogen-related factors when evaluating associations of SNPs involved in the estrogen pathway with MD.

Although no overall association was found between three SNPs (rs598126, rs2010750 and rs676387 in strong linkage disequilibrium) in the *HSD17B1 *gene and MD, we found some associations within strata of BMI and hormonal derivatives used. We observed a decrease in MD with an increasing number of the rare allele of rs598126 SNP among women with higher BMI or past hormonal derivatives users. In contrast, among women who never used hormonal derivatives, MD increased by about 20 cm² or 14% for women who are homozygote for the rare allele (TT for rs598126 or AA for rs2010750) compared to women homozygote for the common allele (CC or GG respectively), and this association was borderline or statistically significant even with a restricted number of women in this stratum. To our knowledge, these SNPs have not been investigated in relation with MD. However, Feigelson et al. who reported no evidence of an association between *HSD17B1 *rs598126 or rs2010750 SNP and overall risk of breast cancer, found that, among tumors that were negative for the estrogen receptors (ER), additional copy of rare alleles was associated with an increased risk of breast cancer [41]. It has been suggested that ER-negative tumors have less estrogenic activity demonstrated by lower levels of estrogen as compared to ER-positive tumors [46]. Since women who never used hormonal derivatives are believed to have less estrogenic activity than women using them [47], results from Feigelson and colleagues on breast cancer risk seem consistent with ours on MD. Little is known about the biology of SNPs in *HSD17B1 *gene, therefore these associations need to be confirmed in further studies.

The evaluation of the relation between *CYP1B1 *rs1056836 SNP and MD did not show a significant association in our overall population of premenopausal women which is consistent with other studies conducted in premenopausal [28,32,33] as well as in postmenopausal [21,22,28] women. However, we observed that, among nulliparous women, carriers of increasing number of G (Val) allele of *CYP1B1 *gene have lower MD. Since nulliparous women have been related to higher estrogenic activity then parous women [48], our results suggest that an increasing number of the Val allele is related to lower MD among women presenting higher estrogenic activity. Although this is the first study to find such association among nulliparous women, Lord et al. observed a similar one among another group of women presenting higher estrogenic activity; postmenopausal hormone (PMH) users [23]. Using data from clinical trials, they reported that among women that used combined estrogen and progesterone for the previous year, the increase in MD was lower for women carrying Val/Val genotype than those carrying Leu/Leu genotype [23]. However, no interaction between *CYP1B1 *rs1056836 SNP and PMH status (current users vs. never or past use) was reported in the cross-sectional study conducted by Haiman et al. [28]. The fact that the activity of the CYP1B1 enzyme is regulated by estrogen may explain why associations of *CYP1B1 *rs1056836 SNP with MD are observed among women presenting higher estrogenic activity. However the biological effect of this polymorphism is still unclear. Data from laboratory studies suggest that the Leu allele might have an increased action in the activation of mammary procarcinogens [49] known to stimulate cell proliferation [50,51]. Conversely, the substitution of leucine to valine in position 432 of the CYP1B1 enzyme has been related to a higher production of 4-OH, increasing the 4-OH/2-OH ratio which could lead to higher mitogenic stimulation of breast cells [49].

Among studies that examined the relation of SNP rs4680 in the *COMT *gene with MD, most of them [22,23,28-31], although not all [31,32], found no association among premenopausal [28-30] or postmenopausal [22,23,28,30,31] women. However, two studies conducted in multiethnic premenopausal women suggested that MD decreased with each additional copy of the A (Met) allele [31,32]. Our findings support a similar association but only among women that had lower estrogenic activity like among those who never used hormonal derivatives or had a late menarche [47,52]. To our knowledge no other study evaluated the possible modifying effect of age at menarche, but three examined the modifying effect of PMH use on the relation between *COMT *rs4680 SNP and MD [23,28,29]. In one study, Haiman and colleagues reported that an increase in the Met allele was associated with a decrease in MD among never and past PMH users, while no association was observed among current users [28]. In another study conducted by the same group, an increase of A (Met) alleles was associated to an increase in MD for current PMH users, and no association was found for never and past users [29]. However, data from a clinical trial showed no association within strata of different PMH uses (estrogen or combined estrogen and progesterone) [23]. Estrogen is known to down-regulate the activity of the COMT enzyme [44] which inactivates by O-methylation the catechol metabolite of estrogen 4-OH. The variant allele of the gene coding for COMT, the Met allele, has been associated to lower enzymatic activity [53,54]. Therefore, the Met allele should be associated with an increased proliferative activity, because of less inactivation of 4-OH in presence of estrogen. It would be biologically reasonable to presume that additional copies of the Met allele would be associated with an increase in MD, particularly among women with increased estrogenic activity. Up to now, observations from our group and others suggest an increase in MD with additional copies of the Met allele among women with higher estrogenic activity (hormonal derivatives users) or a decrease in MD with additional copies of the Met allele among women with lower estrogenic activity (late age at menarche or those who never hormonal derivatives) [28,29].

We evaluated the relation of SNPs in *ERα *or *ERβ *gene with MD among premenopausal women only, and our results showed no significant association or effect modification by estrogen-related factors. Up to now, the association between SNPs in *ER *genes and MD has been inconsistent [22,24,26,27,33]. Crandall et al. found no association between SNP rs9340799 (*ERα*) or rs1256049 (*ERβ*) and MD among premenopausal women but the genotype homozygote for the rare allele of SNP rs2234693 (*ERα*) was related to a higher MD [33]. Among a population composed of premenopausal and postmenopausal women, MD was not related to *ERα *rs2234693 SNP but it had a negative association with the rare allele of rs9340799 SNP [26]. Among postmenopausal women, no association was found for *ERα *rs2234693 [22] or rs2077647 [24] SNP and MD, but the rare allele of rs9340799 SNP was related to a higher MD [24].

This study has some limitations. We cannot exclude that our findings may be due to chance because we evaluated several associations. Type I errors or false-positive results are therefore possible. Population stratification can be a concern in this type of study [55,56]. However, it might not be a problem here because our population was mostly composed (99.7%) of Caucasian women and of over 87.7% French-Canadians [39]. Non-differential misclassification bias from genotyping measurements is possible although it is unlikely in the absence of Hardy-Weinberg disequilibrium and complete concordance for repeated samples. Non-differential misclassification of MD is also possible. However, if present, it should be relatively small and most likely random because all of the mammograms were performed at the same clinic and MD was assessed blindly by one observer whose reliability of reading was shown to be high.

## Conclusion

This study proposes that some SNPs located in the *CYP1B1*, *COMT *or *HSD17B1 *genes are associated with MD among nulliparous women, non-hormonal users, those with late menarche or among women with high BMI. Our findings suggest that modifying effects of estrogen-related factors should be considered when evaluating associations of polymorphisms in estrogen-related genes with premenopausal mammographic density.

## Abbreviations

AD: absolute density; BMI: body mass index; COMT: catechol-O-methyltransferase; CYP1B1: cytochrome P450 1B1; ERα: estrogen receptor alpha; ERβ: estrogen receptor beta; HRT: hormone replacement therapy; HSD17B1: 17β-hydroxysteroid dehydrogenase 1; MD: mammographic density; PD: percent density; PMH: postmenopausal hormone; SD: standard deviation; SNP: single nucleotide polymorphism; 2-OH: 2-hydroxyestrogen; 4-OH: 4-hydroxyestrogen

## Competing interests

The authors declare that they have no competing interests.

## Authors' contributions

ID contributed in the data analysis and interpretation, and wrote the manuscript. CD was involved in the study design, data collection, mammographic breast density assessments, data analysis and interpretation, and manuscript revision. All authors read and approved the final manuscript.

## Pre-publication history

The pre-publication history for this paper can be accessed here:

http://www.biomedcentral.com/1471-2407/10/636/prepub

## Supplementary Material

Additional file 1 **Modifying effect of parity on the association between SNPs and mammographic density**. Click here for file

Additional file 2 **Modifying effect of hormonal derivatives used on the association between SNPs and mammographic density**. Click here for file

Additional file 3 **Modifying effect of age at menarche on the association between SNPs and mammographic density**. Click here for file

Additional file 4 **Modifying effect of body mass index on the association between SNPs and mammographic density**. Click here for file
