# Supplementary material for: Polymorphisms in genes involved in the estrogen pathway and mammographic density
Source: BMC Cancer. 2010 Nov 22;10:636. doi: 10.1186/1471-2407-10-636 (PMC3000407; doi:10.1186/1471-2407-10-636)
Supplement: Additional file 1 — Modifying effect of parity on the association between SNPs and mammographic density. [file 1471-2407-10-636-S1.DOC]

| [**Additional file 1. Modifying effect of parity on the association between SNPs and mammographic density**](../%0DAdditional%20file%201.%20Modifying%20effect%20of%20parity%20on%20the%20association%20between%20SNPs%20and%20mammographic%20density%07%07Gene%07SNP%07%07%07Adjusted%20mean%20mammographic%20densitya%07%07Name%07Reference%20IDb%07Genotype%07N(%25)%07Percent%20density%20(%25)%07Absolute%20density%20(cm2)c%07%07Parity%07%07%07No%07Yes%07No%07Yes%07No%07Yes%07%07ERα%07rs2077647%07TT%0751%20(7.1)%07137%20(19.1)%0744.6%20(39.2-50.1)%0741.8%20(38.4-45.1)%0750.3%20(42.8-58.5)%0746.6%20(42.1-51.4)%07%07%07%07TC%0782%20(11.4)%07270%20(37.6)%0744.6%20(40.3-49.0)%0741.9%20(39.6-44.3)%0746.5%20(40.7-52.7)%0747.4%20(44.2-50.8)%07%07%07%07CC%0739%20(5.4)%07139%20(19.4)%0745.2%20(38.8-51.6)%0742.7%20(39.4-46.0)%0743.4%20(35.4-52.3)%0745.0%20(40.7-49.6)%07%07%07%07Ptrendd%07%07%070.91%070.70%070.24%070.62%07%07%07%07P%20ie%07%07%070.93%070.43%07%07%07rs2234693%07AA%0752%20(7.2)%07157%20(21.7)%0744.0%20(38.5-49.4)%0741.7%20(38.5-44.8)%0750.9%20(43.4-59.1)%0747.6%20(43.3-52.0)%07%07%07%07AG%0787%20(12.0)%07259%20(35.7)%0745.7%20(41.4-49.9)%0741.1%20(38.7-43.5)%0746.6%20(41.0-52.7)%0746.9%20(43.6-50.3)%07%07%07%07GG%0734%20(4.7)%07136%20(18.8)%0744.6%20(37.8-51.4)%0743.3%20(39.9-46.6)%0744.6%20(36.0-54.2)%0744.2%20(39.8-48.8)%07%07%07%07Ptrend%07%07%070.82%070.51%070.28%070.30%07%07%07%07P%20i%07%07%070.91%070.65%07%07%07rs9340799%07AA%0781%20(11.2)%07218%20(30.1)%0744.4%20(40.1-48.8)%0742.2%20(39.6-44.8)%0750.1%20(44.1-56.6)%0749.2%20(45.5-53.0)%07%07%07%07AG%0769%20(9.5)%07252%20(34.8)%0745.0%20(40.2-49.7)%0740.9%20(38.4-43.3)%0743.5%20(37.5-50.1)%0744.9%20(41.7-48.3)%07%07%07%07GG%0723%20(3.2)%0782%20(11.3)%0747.3%20(39.1-55.6)%0744.0%20(39.7-48.3)%0751.3%20(40.1-64.0)%0744.7%20(39.2-50.7)%07%07%07%07Ptrend%07%07%070.58%070.76%070.65%070.11%07%07%07%07P%20i%07%07%070.74%070.70%07%07%07rs2228480%07CC%07126%20(17.4)%07376%20(51.8)%0744.8%20(41.3-48.3)%0741.1%20(39.1-43.1)%0748.1%20(43.4-53.2)%0745.5%20(42.8-48.2)%07%07%07%07CT%0746%20(6.3)%07157%20(21.6)%0745.2%20(39.4-51.0)%0743.0%20(39.9-46.2)%0744.5%20(37.1-52.7)%0748.1%20(43.9-52.6)%07%07%07%07TT%072%20(0.3)%0719%20(2.6)%0716.9%20(-10.7-44.4)%0748.9%20(39.9-57.8)%0729.2%20(8.6-66.9)%0757.3%20(44.4-72.0)%07%07%07%07Ptrend%07%07%070.51%070.09%070.27%070.08%07%07%07%07P%20i%07%07%070.18%070.08%07%07ERβ%07rs3829768%07TT%07173%20(23.9)%07550%20(75.9)%0744.8%20(41.8-47.8)%0741.9%20(40.2-43.5)%0747.2%20(43.1-51.5)%0746.5%20(44.3-48.8)%07%07%07%07TC%071%20(0.1)%071%20(0.1)%07-8.2%20(-47.1-30.7)%0741.6%20(2.4-80.8)%076.0%20(12.3-33.8)%0792.5%20(31.7-187.9)%07%07%07%07Ptrend%07%07%070.008%070.99%070.01%070.17%07%07%07%07P%20i%07%07%070.06%070.006%07%07%07rs1256049%07GG%07162%20(22.3)%07517%20(71.1)%0745.4%20(42.3-48.5)%0741.9%20(40.2-43.6)%0748.0%20(43.7-52.4)%0746.6%20(44.2-49.0)%07%07%07%07GA%0712%20(1.7)%0736%20(5.0)%0737.9%20(26.6-49.2)%0740.8%20(34.3-47.4)%0741.0%20(27.9-56.9)%0746.1%20(37.6-55.6)%07%07%07%07AA%070%070%07%07%07%07%07%07%07%07Ptrend%07%07%070.21%070.76%070.38%070.92%07%07%07%07P%20i%07%07%070.35%070.48%07%07HSD17B1%07rs676387%07GG%0788%20(12.1)%07296%20(40.7)%0746.0%20(41.8-50.2)%0740.4%20(38.2-42.7)%0745.1%20(39.6-51.0)%0745.2%20(42.2-48.4)%07%07%07%07GT%0775%20(10.3)%07212%20(29.2)%0742.8%20(38.3-47.4)%0743.8%20(41.1-46.4)%0748.7%20(42.5-55.2)%0748.4%20(44.7-52.2)%07%07%07%07TT%0711%20(1.5)%0745%20(6.2)%0743.4%20(31.7-55.2)%0742.1%20(36.3-47.9)%0748.8%20(33.8-67.0)%0746.6%20(39.0-55.0)%07%07%07%07Ptrend%07%07%070.37%070.16%070.42%070.34%07%07%07%07P%20i%07%07%070.14%070.80%07%07%07rs598126%07CC%0742%20(5.8)%07149%20(20.6)%0744.5%20(38.4-50.5)%0742.2%20(39.0-45.4)%0751.1%20(42.8-60.3)%0748.1%20(43.8-52.7)%07%07%07%07CT%0787%20(12.1)%07263%20(36.4)%0743.5%20(39.3-47.7)%0743.0%20(40.6-45.4)%0745.9%20(40.4-51.9)%0746.8%20(43.5-50.1)%07%07%07%07TT%0744%20(6.1)%07137%20(19.0)%0746.8%20(40.9-52.7)%0738.9%20(35.5-42.3)%0745.0%20(37.4-53.4)%0743.8%20(39.4-48.4)%07%07%07%07Ptrend%07%07%070.58%070.18%070.31%070.18%07%07%07%07P%20i%07%07%070.26%070.81%07%07%07rs2010750%07GG%0747%20(6.5)%07186%20(25.7)%0744.5%20(38.8-50.2)%0742.4%20(39.5-45.2)%0750.8%20(42.9-59.4)%0747.1%20(43.2-51.1)%07%07%07%07GA%0789%20(12.3)%07258%20(35.6)%0743.9%20(39.7-48.1)%0742.8%20(40.4-45.2)%0746.3%20(40.8-52.2)%0747.6%20(44.3-51.1)%07%07%07%07AA%0737%20(5.1)%07107%20(14.8)%0746.6%20(40.2-53.1)%0738.9%20(35.1-42.7)%0743.9%20(35.7-52.9)%0743.7%20(38.8-49.0)%07%07%07%07Ptrend%07%07%070.66%070.22%070.25%070.39%07%07%07%07P%20i%07%07%070.33%070.55%07%07COMT%07rs4680%07GG%0744%20(6.1)%07143%20(19.7)%0743.1%20(37.1-49.1)%0742.0%20(38.7-45.3)%0743.3%20(35.7-51.7)%0748.8%20(44.3-53.6)%07%07%07%07GA%0796%20(13.2)%07275%20(37.9)%0745.3%20(41.3-49.3)%0743.3%20(41.0-45.7)%0746.4%20(41.1-52.1)%0746.7%20(43.5-50.1)%07%07%07%07AA%0734%20(4.7)%07133%20(18.3)%0743.4%20(36.7-50.0)%0738.6%20(35.2-42.0)%0752.6%20(43.3-62.9)%0743.6%20(39.3-48.2)%07%07%07%07Ptrend%07%07%070.93%070.17%070.15%070.12%07%07%07%07P%20i%07%07%070.47%070.05%07%07CYP1B1%07rs1056836%07CC%0753%20(7.3)%07163%20(22.4)%0746.6%20(41.1-52.1)%0742.8%20(39.7-45.8)%0754.2%20(46.4-62.7)%0747.2%20(43.1-51.5)%07%07%07%07CG%0786%20(11.8)%07282%20(38.8)%0746.3%20(42.0-50.5)%0741.8%20(39.5-44.1)%0747.0%20(41.3-53.0)%0746.4%20(43.3-49.6)%07%07%07%07GG%0735%20(4.8)%07108%20(14.9)%0736.9%20(30.3-43.5)%0740.6%20(36.8-44.3)%0737.0%20(29.5-45.4)%0745.5%20(40.6-50.7)%07%07%07%07Ptrend%07%07%070.04%070.38%070.004%070.62%07%07%07%07P%20i%07%07%070.19%070.02%07%07a%20Analyses%20are%20adjusted%20for%20age%20at%20mammography,%20body%20mass%20index,%20waist-to-hip%20ratio,%20height,%20age%20at%20menarche,%20age%20at%20first%20birth,%20number%20of%20full-term%20pregnancies,%20breastfeeding,%20number%20of%20breast%20biopsies,%20family%20history%20of%20breast%20cancer,%20past%20contraceptive%20and%20hormone%20replacement%20therapy%20uses,%20smoking%20status,%20energy%20and%20alcohol%20intakes,%20physical%20activity%20and%20education%20when%20applicable.%20Except%20for%20modifying%20effect%20of%20parity,%20models%20were%20also%20adjusted%20for%20age%20at%20first%20birth,%20number%20of%20full-term%20pregnancies%20and%20breastfeeding.%0Db%20%20SNPs:%20single%20nucleotide%20polymorphisms.%20They%20are%20identified%20by%20their%20dbSNP%20accession%20number%20at%20http://www.ncbi.nlm.nih.gov/SNP/.%0Dc%20Means%20of%20absolute%20density%20are%20presented%20as%20back-transformed%20values.%0Dd%20%20P%20value%20is%20the%20p%20trend,%20testing%20genotype%20dosage,%20number%20of%20copies%20of%20the%20rare%20allele%20entered%20as%200,%201,%202%20and%20mammographic%20density%20entered%20as%20a%20continuous%20variable.%0De%20%20P%20value%20for%20interaction%20between%20the%20variable%20(parity,%20hormonal%20derivative%20used,%20age%20at%20menarche%20or%20body%20mass%20index)%20and%20the%20genotype%20dosage%20%0Dfrom%20linear%20regression.%07%07) | | | | | | | | | |
| --- | --- | --- | --- | --- | --- | --- | --- | --- | --- |
| [Gene](../%0DAdditional%20file%201.%20Modifying%20effect%20of%20parity%20on%20the%20association%20between%20SNPs%20and%20mammographic%20density%07%07Gene%07SNP%07%07%07Adjusted%20mean%20mammographic%20densitya%07%07Name%07Reference%20IDb%07Genotype%07N(%25)%07Percent%20density%20(%25)%07Absolute%20density%20(cm2)c%07%07Parity%07%07%07No%07Yes%07No%07Yes%07No%07Yes%07%07ERα%07rs2077647%07TT%0751%20(7.1)%07137%20(19.1)%0744.6%20(39.2-50.1)%0741.8%20(38.4-45.1)%0750.3%20(42.8-58.5)%0746.6%20(42.1-51.4)%07%07%07%07TC%0782%20(11.4)%07270%20(37.6)%0744.6%20(40.3-49.0)%0741.9%20(39.6-44.3)%0746.5%20(40.7-52.7)%0747.4%20(44.2-50.8)%07%07%07%07CC%0739%20(5.4)%07139%20(19.4)%0745.2%20(38.8-51.6)%0742.7%20(39.4-46.0)%0743.4%20(35.4-52.3)%0745.0%20(40.7-49.6)%07%07%07%07Ptrendd%07%07%070.91%070.70%070.24%070.62%07%07%07%07P%20ie%07%07%070.93%070.43%07%07%07rs2234693%07AA%0752%20(7.2)%07157%20(21.7)%0744.0%20(38.5-49.4)%0741.7%20(38.5-44.8)%0750.9%20(43.4-59.1)%0747.6%20(43.3-52.0)%07%07%07%07AG%0787%20(12.0)%07259%20(35.7)%0745.7%20(41.4-49.9)%0741.1%20(38.7-43.5)%0746.6%20(41.0-52.7)%0746.9%20(43.6-50.3)%07%07%07%07GG%0734%20(4.7)%07136%20(18.8)%0744.6%20(37.8-51.4)%0743.3%20(39.9-46.6)%0744.6%20(36.0-54.2)%0744.2%20(39.8-48.8)%07%07%07%07Ptrend%07%07%070.82%070.51%070.28%070.30%07%07%07%07P%20i%07%07%070.91%070.65%07%07%07rs9340799%07AA%0781%20(11.2)%07218%20(30.1)%0744.4%20(40.1-48.8)%0742.2%20(39.6-44.8)%0750.1%20(44.1-56.6)%0749.2%20(45.5-53.0)%07%07%07%07AG%0769%20(9.5)%07252%20(34.8)%0745.0%20(40.2-49.7)%0740.9%20(38.4-43.3)%0743.5%20(37.5-50.1)%0744.9%20(41.7-48.3)%07%07%07%07GG%0723%20(3.2)%0782%20(11.3)%0747.3%20(39.1-55.6)%0744.0%20(39.7-48.3)%0751.3%20(40.1-64.0)%0744.7%20(39.2-50.7)%07%07%07%07Ptrend%07%07%070.58%070.76%070.65%070.11%07%07%07%07P%20i%07%07%070.74%070.70%07%07%07rs2228480%07CC%07126%20(17.4)%07376%20(51.8)%0744.8%20(41.3-48.3)%0741.1%20(39.1-43.1)%0748.1%20(43.4-53.2)%0745.5%20(42.8-48.2)%07%07%07%07CT%0746%20(6.3)%07157%20(21.6)%0745.2%20(39.4-51.0)%0743.0%20(39.9-46.2)%0744.5%20(37.1-52.7)%0748.1%20(43.9-52.6)%07%07%07%07TT%072%20(0.3)%0719%20(2.6)%0716.9%20(-10.7-44.4)%0748.9%20(39.9-57.8)%0729.2%20(8.6-66.9)%0757.3%20(44.4-72.0)%07%07%07%07Ptrend%07%07%070.51%070.09%070.27%070.08%07%07%07%07P%20i%07%07%070.18%070.08%07%07ERβ%07rs3829768%07TT%07173%20(23.9)%07550%20(75.9)%0744.8%20(41.8-47.8)%0741.9%20(40.2-43.5)%0747.2%20(43.1-51.5)%0746.5%20(44.3-48.8)%07%07%07%07TC%071%20(0.1)%071%20(0.1)%07-8.2%20(-47.1-30.7)%0741.6%20(2.4-80.8)%076.0%20(12.3-33.8)%0792.5%20(31.7-187.9)%07%07%07%07Ptrend%07%07%070.008%070.99%070.01%070.17%07%07%07%07P%20i%07%07%070.06%070.006%07%07%07rs1256049%07GG%07162%20(22.3)%07517%20(71.1)%0745.4%20(42.3-48.5)%0741.9%20(40.2-43.6)%0748.0%20(43.7-52.4)%0746.6%20(44.2-49.0)%07%07%07%07GA%0712%20(1.7)%0736%20(5.0)%0737.9%20(26.6-49.2)%0740.8%20(34.3-47.4)%0741.0%20(27.9-56.9)%0746.1%20(37.6-55.6)%07%07%07%07AA%070%070%07%07%07%07%07%07%07%07Ptrend%07%07%070.21%070.76%070.38%070.92%07%07%07%07P%20i%07%07%070.35%070.48%07%07HSD17B1%07rs676387%07GG%0788%20(12.1)%07296%20(40.7)%0746.0%20(41.8-50.2)%0740.4%20(38.2-42.7)%0745.1%20(39.6-51.0)%0745.2%20(42.2-48.4)%07%07%07%07GT%0775%20(10.3)%07212%20(29.2)%0742.8%20(38.3-47.4)%0743.8%20(41.1-46.4)%0748.7%20(42.5-55.2)%0748.4%20(44.7-52.2)%07%07%07%07TT%0711%20(1.5)%0745%20(6.2)%0743.4%20(31.7-55.2)%0742.1%20(36.3-47.9)%0748.8%20(33.8-67.0)%0746.6%20(39.0-55.0)%07%07%07%07Ptrend%07%07%070.37%070.16%070.42%070.34%07%07%07%07P%20i%07%07%070.14%070.80%07%07%07rs598126%07CC%0742%20(5.8)%07149%20(20.6)%0744.5%20(38.4-50.5)%0742.2%20(39.0-45.4)%0751.1%20(42.8-60.3)%0748.1%20(43.8-52.7)%07%07%07%07CT%0787%20(12.1)%07263%20(36.4)%0743.5%20(39.3-47.7)%0743.0%20(40.6-45.4)%0745.9%20(40.4-51.9)%0746.8%20(43.5-50.1)%07%07%07%07TT%0744%20(6.1)%07137%20(19.0)%0746.8%20(40.9-52.7)%0738.9%20(35.5-42.3)%0745.0%20(37.4-53.4)%0743.8%20(39.4-48.4)%07%07%07%07Ptrend%07%07%070.58%070.18%070.31%070.18%07%07%07%07P%20i%07%07%070.26%070.81%07%07%07rs2010750%07GG%0747%20(6.5)%07186%20(25.7)%0744.5%20(38.8-50.2)%0742.4%20(39.5-45.2)%0750.8%20(42.9-59.4)%0747.1%20(43.2-51.1)%07%07%07%07GA%0789%20(12.3)%07258%20(35.6)%0743.9%20(39.7-48.1)%0742.8%20(40.4-45.2)%0746.3%20(40.8-52.2)%0747.6%20(44.3-51.1)%07%07%07%07AA%0737%20(5.1)%07107%20(14.8)%0746.6%20(40.2-53.1)%0738.9%20(35.1-42.7)%0743.9%20(35.7-52.9)%0743.7%20(38.8-49.0)%07%07%07%07Ptrend%07%07%070.66%070.22%070.25%070.39%07%07%07%07P%20i%07%07%070.33%070.55%07%07COMT%07rs4680%07GG%0744%20(6.1)%07143%20(19.7)%0743.1%20(37.1-49.1)%0742.0%20(38.7-45.3)%0743.3%20(35.7-51.7)%0748.8%20(44.3-53.6)%07%07%07%07GA%0796%20(13.2)%07275%20(37.9)%0745.3%20(41.3-49.3)%0743.3%20(41.0-45.7)%0746.4%20(41.1-52.1)%0746.7%20(43.5-50.1)%07%07%07%07AA%0734%20(4.7)%07133%20(18.3)%0743.4%20(36.7-50.0)%0738.6%20(35.2-42.0)%0752.6%20(43.3-62.9)%0743.6%20(39.3-48.2)%07%07%07%07Ptrend%07%07%070.93%070.17%070.15%070.12%07%07%07%07P%20i%07%07%070.47%070.05%07%07CYP1B1%07rs1056836%07CC%0753%20(7.3)%07163%20(22.4)%0746.6%20(41.1-52.1)%0742.8%20(39.7-45.8)%0754.2%20(46.4-62.7)%0747.2%20(43.1-51.5)%07%07%07%07CG%0786%20(11.8)%07282%20(38.8)%0746.3%20(42.0-50.5)%0741.8%20(39.5-44.1)%0747.0%20(41.3-53.0)%0746.4%20(43.3-49.6)%07%07%07%07GG%0735%20(4.8)%07108%20(14.9)%0736.9%20(30.3-43.5)%0740.6%20(36.8-44.3)%0737.0%20(29.5-45.4)%0745.5%20(40.6-50.7)%07%07%07%07Ptrend%07%07%070.04%070.38%070.004%070.62%07%07%07%07P%20i%07%07%070.19%070.02%07%07a%20Analyses%20are%20adjusted%20for%20age%20at%20mammography,%20body%20mass%20index,%20waist-to-hip%20ratio,%20height,%20age%20at%20menarche,%20age%20at%20first%20birth,%20number%20of%20full-term%20pregnancies,%20breastfeeding,%20number%20of%20breast%20biopsies,%20family%20history%20of%20breast%20cancer,%20past%20contraceptive%20and%20hormone%20replacement%20therapy%20uses,%20smoking%20status,%20energy%20and%20alcohol%20intakes,%20physical%20activity%20and%20education%20when%20applicable.%20Except%20for%20modifying%20effect%20of%20parity,%20models%20were%20also%20adjusted%20for%20age%20at%20first%20birth,%20number%20of%20full-term%20pregnancies%20and%20breastfeeding.%0Db%20%20SNPs:%20single%20nucleotide%20polymorphisms.%20They%20are%20identified%20by%20their%20dbSNP%20accession%20number%20at%20http://www.ncbi.nlm.nih.gov/SNP/.%0Dc%20Means%20of%20absolute%20density%20are%20presented%20as%20back-transformed%20values.%0Dd%20%20P%20value%20is%20the%20p%20trend,%20testing%20genotype%20dosage,%20number%20of%20copies%20of%20the%20rare%20allele%20entered%20as%200,%201,%202%20and%20mammographic%20density%20entered%20as%20a%20continuous%20variable.%0De%20%20P%20value%20for%20interaction%20between%20the%20variable%20(parity,%20hormonal%20derivative%20used,%20age%20at%20menarche%20or%20body%20mass%20index)%20and%20the%20genotype%20dosage%20%0Dfrom%20linear%20regression.%07%07) | SNP |  |  | | | Adjusted mean mammographic density (95% CI)^a^ | | | |
| [name](../%0DAdditional%20file%201.%20Modifying%20effect%20of%20parity%20on%20the%20association%20between%20SNPs%20and%20mammographic%20density%07%07Gene%07SNP%07%07%07Adjusted%20mean%20mammographic%20densitya%07%07Name%07Reference%20IDb%07Genotype%07N(%25)%07Percent%20density%20(%25)%07Absolute%20density%20(cm2)c%07%07Parity%07%07%07No%07Yes%07No%07Yes%07No%07Yes%07%07ERα%07rs2077647%07TT%0751%20(7.1)%07137%20(19.1)%0744.6%20(39.2-50.1)%0741.8%20(38.4-45.1)%0750.3%20(42.8-58.5)%0746.6%20(42.1-51.4)%07%07%07%07TC%0782%20(11.4)%07270%20(37.6)%0744.6%20(40.3-49.0)%0741.9%20(39.6-44.3)%0746.5%20(40.7-52.7)%0747.4%20(44.2-50.8)%07%07%07%07CC%0739%20(5.4)%07139%20(19.4)%0745.2%20(38.8-51.6)%0742.7%20(39.4-46.0)%0743.4%20(35.4-52.3)%0745.0%20(40.7-49.6)%07%07%07%07Ptrendd%07%07%070.91%070.70%070.24%070.62%07%07%07%07P%20ie%07%07%070.93%070.43%07%07%07rs2234693%07AA%0752%20(7.2)%07157%20(21.7)%0744.0%20(38.5-49.4)%0741.7%20(38.5-44.8)%0750.9%20(43.4-59.1)%0747.6%20(43.3-52.0)%07%07%07%07AG%0787%20(12.0)%07259%20(35.7)%0745.7%20(41.4-49.9)%0741.1%20(38.7-43.5)%0746.6%20(41.0-52.7)%0746.9%20(43.6-50.3)%07%07%07%07GG%0734%20(4.7)%07136%20(18.8)%0744.6%20(37.8-51.4)%0743.3%20(39.9-46.6)%0744.6%20(36.0-54.2)%0744.2%20(39.8-48.8)%07%07%07%07Ptrend%07%07%070.82%070.51%070.28%070.30%07%07%07%07P%20i%07%07%070.91%070.65%07%07%07rs9340799%07AA%0781%20(11.2)%07218%20(30.1)%0744.4%20(40.1-48.8)%0742.2%20(39.6-44.8)%0750.1%20(44.1-56.6)%0749.2%20(45.5-53.0)%07%07%07%07AG%0769%20(9.5)%07252%20(34.8)%0745.0%20(40.2-49.7)%0740.9%20(38.4-43.3)%0743.5%20(37.5-50.1)%0744.9%20(41.7-48.3)%07%07%07%07GG%0723%20(3.2)%0782%20(11.3)%0747.3%20(39.1-55.6)%0744.0%20(39.7-48.3)%0751.3%20(40.1-64.0)%0744.7%20(39.2-50.7)%07%07%07%07Ptrend%07%07%070.58%070.76%070.65%070.11%07%07%07%07P%20i%07%07%070.74%070.70%07%07%07rs2228480%07CC%07126%20(17.4)%07376%20(51.8)%0744.8%20(41.3-48.3)%0741.1%20(39.1-43.1)%0748.1%20(43.4-53.2)%0745.5%20(42.8-48.2)%07%07%07%07CT%0746%20(6.3)%07157%20(21.6)%0745.2%20(39.4-51.0)%0743.0%20(39.9-46.2)%0744.5%20(37.1-52.7)%0748.1%20(43.9-52.6)%07%07%07%07TT%072%20(0.3)%0719%20(2.6)%0716.9%20(-10.7-44.4)%0748.9%20(39.9-57.8)%0729.2%20(8.6-66.9)%0757.3%20(44.4-72.0)%07%07%07%07Ptrend%07%07%070.51%070.09%070.27%070.08%07%07%07%07P%20i%07%07%070.18%070.08%07%07ERβ%07rs3829768%07TT%07173%20(23.9)%07550%20(75.9)%0744.8%20(41.8-47.8)%0741.9%20(40.2-43.5)%0747.2%20(43.1-51.5)%0746.5%20(44.3-48.8)%07%07%07%07TC%071%20(0.1)%071%20(0.1)%07-8.2%20(-47.1-30.7)%0741.6%20(2.4-80.8)%076.0%20(12.3-33.8)%0792.5%20(31.7-187.9)%07%07%07%07Ptrend%07%07%070.008%070.99%070.01%070.17%07%07%07%07P%20i%07%07%070.06%070.006%07%07%07rs1256049%07GG%07162%20(22.3)%07517%20(71.1)%0745.4%20(42.3-48.5)%0741.9%20(40.2-43.6)%0748.0%20(43.7-52.4)%0746.6%20(44.2-49.0)%07%07%07%07GA%0712%20(1.7)%0736%20(5.0)%0737.9%20(26.6-49.2)%0740.8%20(34.3-47.4)%0741.0%20(27.9-56.9)%0746.1%20(37.6-55.6)%07%07%07%07AA%070%070%07%07%07%07%07%07%07%07Ptrend%07%07%070.21%070.76%070.38%070.92%07%07%07%07P%20i%07%07%070.35%070.48%07%07HSD17B1%07rs676387%07GG%0788%20(12.1)%07296%20(40.7)%0746.0%20(41.8-50.2)%0740.4%20(38.2-42.7)%0745.1%20(39.6-51.0)%0745.2%20(42.2-48.4)%07%07%07%07GT%0775%20(10.3)%07212%20(29.2)%0742.8%20(38.3-47.4)%0743.8%20(41.1-46.4)%0748.7%20(42.5-55.2)%0748.4%20(44.7-52.2)%07%07%07%07TT%0711%20(1.5)%0745%20(6.2)%0743.4%20(31.7-55.2)%0742.1%20(36.3-47.9)%0748.8%20(33.8-67.0)%0746.6%20(39.0-55.0)%07%07%07%07Ptrend%07%07%070.37%070.16%070.42%070.34%07%07%07%07P%20i%07%07%070.14%070.80%07%07%07rs598126%07CC%0742%20(5.8)%07149%20(20.6)%0744.5%20(38.4-50.5)%0742.2%20(39.0-45.4)%0751.1%20(42.8-60.3)%0748.1%20(43.8-52.7)%07%07%07%07CT%0787%20(12.1)%07263%20(36.4)%0743.5%20(39.3-47.7)%0743.0%20(40.6-45.4)%0745.9%20(40.4-51.9)%0746.8%20(43.5-50.1)%07%07%07%07TT%0744%20(6.1)%07137%20(19.0)%0746.8%20(40.9-52.7)%0738.9%20(35.5-42.3)%0745.0%20(37.4-53.4)%0743.8%20(39.4-48.4)%07%07%07%07Ptrend%07%07%070.58%070.18%070.31%070.18%07%07%07%07P%20i%07%07%070.26%070.81%07%07%07rs2010750%07GG%0747%20(6.5)%07186%20(25.7)%0744.5%20(38.8-50.2)%0742.4%20(39.5-45.2)%0750.8%20(42.9-59.4)%0747.1%20(43.2-51.1)%07%07%07%07GA%0789%20(12.3)%07258%20(35.6)%0743.9%20(39.7-48.1)%0742.8%20(40.4-45.2)%0746.3%20(40.8-52.2)%0747.6%20(44.3-51.1)%07%07%07%07AA%0737%20(5.1)%07107%20(14.8)%0746.6%20(40.2-53.1)%0738.9%20(35.1-42.7)%0743.9%20(35.7-52.9)%0743.7%20(38.8-49.0)%07%07%07%07Ptrend%07%07%070.66%070.22%070.25%070.39%07%07%07%07P%20i%07%07%070.33%070.55%07%07COMT%07rs4680%07GG%0744%20(6.1)%07143%20(19.7)%0743.1%20(37.1-49.1)%0742.0%20(38.7-45.3)%0743.3%20(35.7-51.7)%0748.8%20(44.3-53.6)%07%07%07%07GA%0796%20(13.2)%07275%20(37.9)%0745.3%20(41.3-49.3)%0743.3%20(41.0-45.7)%0746.4%20(41.1-52.1)%0746.7%20(43.5-50.1)%07%07%07%07AA%0734%20(4.7)%07133%20(18.3)%0743.4%20(36.7-50.0)%0738.6%20(35.2-42.0)%0752.6%20(43.3-62.9)%0743.6%20(39.3-48.2)%07%07%07%07Ptrend%07%07%070.93%070.17%070.15%070.12%07%07%07%07P%20i%07%07%070.47%070.05%07%07CYP1B1%07rs1056836%07CC%0753%20(7.3)%07163%20(22.4)%0746.6%20(41.1-52.1)%0742.8%20(39.7-45.8)%0754.2%20(46.4-62.7)%0747.2%20(43.1-51.5)%07%07%07%07CG%0786%20(11.8)%07282%20(38.8)%0746.3%20(42.0-50.5)%0741.8%20(39.5-44.1)%0747.0%20(41.3-53.0)%0746.4%20(43.3-49.6)%07%07%07%07GG%0735%20(4.8)%07108%20(14.9)%0736.9%20(30.3-43.5)%0740.6%20(36.8-44.3)%0737.0%20(29.5-45.4)%0745.5%20(40.6-50.7)%07%07%07%07Ptrend%07%07%070.04%070.38%070.004%070.62%07%07%07%07P%20i%07%07%070.19%070.02%07%07a%20Analyses%20are%20adjusted%20for%20age%20at%20mammography,%20body%20mass%20index,%20waist-to-hip%20ratio,%20height,%20age%20at%20menarche,%20age%20at%20first%20birth,%20number%20of%20full-term%20pregnancies,%20breastfeeding,%20number%20of%20breast%20biopsies,%20family%20history%20of%20breast%20cancer,%20past%20contraceptive%20and%20hormone%20replacement%20therapy%20uses,%20smoking%20status,%20energy%20and%20alcohol%20intakes,%20physical%20activity%20and%20education%20when%20applicable.%20Except%20for%20modifying%20effect%20of%20parity,%20models%20were%20also%20adjusted%20for%20age%20at%20first%20birth,%20number%20of%20full-term%20pregnancies%20and%20breastfeeding.%0Db%20%20SNPs:%20single%20nucleotide%20polymorphisms.%20They%20are%20identified%20by%20their%20dbSNP%20accession%20number%20at%20http://www.ncbi.nlm.nih.gov/SNP/.%0Dc%20Means%20of%20absolute%20density%20are%20presented%20as%20back-transformed%20values.%0Dd%20%20P%20value%20is%20the%20p%20trend,%20testing%20genotype%20dosage,%20number%20of%20copies%20of%20the%20rare%20allele%20entered%20as%200,%201,%202%20and%20mammographic%20density%20entered%20as%20a%20continuous%20variable.%0De%20%20P%20value%20for%20interaction%20between%20the%20variable%20(parity,%20hormonal%20derivative%20used,%20age%20at%20menarche%20or%20body%20mass%20index)%20and%20the%20genotype%20dosage%20%0Dfrom%20linear%20regression.%07%07) | reference ID^b^ | Genotype | N(%) | | | Percent density (%) | | Absolute density (cm^2^)^c^ | |
| [**Parity :**](../%0DAdditional%20file%201.%20Modifying%20effect%20of%20parity%20on%20the%20association%20between%20SNPs%20and%20mammographic%20density%07%07Gene%07SNP%07%07%07Adjusted%20mean%20mammographic%20densitya%07%07Name%07Reference%20IDb%07Genotype%07N(%25)%07Percent%20density%20(%25)%07Absolute%20density%20(cm2)c%07%07Parity%07%07%07No%07Yes%07No%07Yes%07No%07Yes%07%07ERα%07rs2077647%07TT%0751%20(7.1)%07137%20(19.1)%0744.6%20(39.2-50.1)%0741.8%20(38.4-45.1)%0750.3%20(42.8-58.5)%0746.6%20(42.1-51.4)%07%07%07%07TC%0782%20(11.4)%07270%20(37.6)%0744.6%20(40.3-49.0)%0741.9%20(39.6-44.3)%0746.5%20(40.7-52.7)%0747.4%20(44.2-50.8)%07%07%07%07CC%0739%20(5.4)%07139%20(19.4)%0745.2%20(38.8-51.6)%0742.7%20(39.4-46.0)%0743.4%20(35.4-52.3)%0745.0%20(40.7-49.6)%07%07%07%07Ptrendd%07%07%070.91%070.70%070.24%070.62%07%07%07%07P%20ie%07%07%070.93%070.43%07%07%07rs2234693%07AA%0752%20(7.2)%07157%20(21.7)%0744.0%20(38.5-49.4)%0741.7%20(38.5-44.8)%0750.9%20(43.4-59.1)%0747.6%20(43.3-52.0)%07%07%07%07AG%0787%20(12.0)%07259%20(35.7)%0745.7%20(41.4-49.9)%0741.1%20(38.7-43.5)%0746.6%20(41.0-52.7)%0746.9%20(43.6-50.3)%07%07%07%07GG%0734%20(4.7)%07136%20(18.8)%0744.6%20(37.8-51.4)%0743.3%20(39.9-46.6)%0744.6%20(36.0-54.2)%0744.2%20(39.8-48.8)%07%07%07%07Ptrend%07%07%070.82%070.51%070.28%070.30%07%07%07%07P%20i%07%07%070.91%070.65%07%07%07rs9340799%07AA%0781%20(11.2)%07218%20(30.1)%0744.4%20(40.1-48.8)%0742.2%20(39.6-44.8)%0750.1%20(44.1-56.6)%0749.2%20(45.5-53.0)%07%07%07%07AG%0769%20(9.5)%07252%20(34.8)%0745.0%20(40.2-49.7)%0740.9%20(38.4-43.3)%0743.5%20(37.5-50.1)%0744.9%20(41.7-48.3)%07%07%07%07GG%0723%20(3.2)%0782%20(11.3)%0747.3%20(39.1-55.6)%0744.0%20(39.7-48.3)%0751.3%20(40.1-64.0)%0744.7%20(39.2-50.7)%07%07%07%07Ptrend%07%07%070.58%070.76%070.65%070.11%07%07%07%07P%20i%07%07%070.74%070.70%07%07%07rs2228480%07CC%07126%20(17.4)%07376%20(51.8)%0744.8%20(41.3-48.3)%0741.1%20(39.1-43.1)%0748.1%20(43.4-53.2)%0745.5%20(42.8-48.2)%07%07%07%07CT%0746%20(6.3)%07157%20(21.6)%0745.2%20(39.4-51.0)%0743.0%20(39.9-46.2)%0744.5%20(37.1-52.7)%0748.1%20(43.9-52.6)%07%07%07%07TT%072%20(0.3)%0719%20(2.6)%0716.9%20(-10.7-44.4)%0748.9%20(39.9-57.8)%0729.2%20(8.6-66.9)%0757.3%20(44.4-72.0)%07%07%07%07Ptrend%07%07%070.51%070.09%070.27%070.08%07%07%07%07P%20i%07%07%070.18%070.08%07%07ERβ%07rs3829768%07TT%07173%20(23.9)%07550%20(75.9)%0744.8%20(41.8-47.8)%0741.9%20(40.2-43.5)%0747.2%20(43.1-51.5)%0746.5%20(44.3-48.8)%07%07%07%07TC%071%20(0.1)%071%20(0.1)%07-8.2%20(-47.1-30.7)%0741.6%20(2.4-80.8)%076.0%20(12.3-33.8)%0792.5%20(31.7-187.9)%07%07%07%07Ptrend%07%07%070.008%070.99%070.01%070.17%07%07%07%07P%20i%07%07%070.06%070.006%07%07%07rs1256049%07GG%07162%20(22.3)%07517%20(71.1)%0745.4%20(42.3-48.5)%0741.9%20(40.2-43.6)%0748.0%20(43.7-52.4)%0746.6%20(44.2-49.0)%07%07%07%07GA%0712%20(1.7)%0736%20(5.0)%0737.9%20(26.6-49.2)%0740.8%20(34.3-47.4)%0741.0%20(27.9-56.9)%0746.1%20(37.6-55.6)%07%07%07%07AA%070%070%07%07%07%07%07%07%07%07Ptrend%07%07%070.21%070.76%070.38%070.92%07%07%07%07P%20i%07%07%070.35%070.48%07%07HSD17B1%07rs676387%07GG%0788%20(12.1)%07296%20(40.7)%0746.0%20(41.8-50.2)%0740.4%20(38.2-42.7)%0745.1%20(39.6-51.0)%0745.2%20(42.2-48.4)%07%07%07%07GT%0775%20(10.3)%07212%20(29.2)%0742.8%20(38.3-47.4)%0743.8%20(41.1-46.4)%0748.7%20(42.5-55.2)%0748.4%20(44.7-52.2)%07%07%07%07TT%0711%20(1.5)%0745%20(6.2)%0743.4%20(31.7-55.2)%0742.1%20(36.3-47.9)%0748.8%20(33.8-67.0)%0746.6%20(39.0-55.0)%07%07%07%07Ptrend%07%07%070.37%070.16%070.42%070.34%07%07%07%07P%20i%07%07%070.14%070.80%07%07%07rs598126%07CC%0742%20(5.8)%07149%20(20.6)%0744.5%20(38.4-50.5)%0742.2%20(39.0-45.4)%0751.1%20(42.8-60.3)%0748.1%20(43.8-52.7)%07%07%07%07CT%0787%20(12.1)%07263%20(36.4)%0743.5%20(39.3-47.7)%0743.0%20(40.6-45.4)%0745.9%20(40.4-51.9)%0746.8%20(43.5-50.1)%07%07%07%07TT%0744%20(6.1)%07137%20(19.0)%0746.8%20(40.9-52.7)%0738.9%20(35.5-42.3)%0745.0%20(37.4-53.4)%0743.8%20(39.4-48.4)%07%07%07%07Ptrend%07%07%070.58%070.18%070.31%070.18%07%07%07%07P%20i%07%07%070.26%070.81%07%07%07rs2010750%07GG%0747%20(6.5)%07186%20(25.7)%0744.5%20(38.8-50.2)%0742.4%20(39.5-45.2)%0750.8%20(42.9-59.4)%0747.1%20(43.2-51.1)%07%07%07%07GA%0789%20(12.3)%07258%20(35.6)%0743.9%20(39.7-48.1)%0742.8%20(40.4-45.2)%0746.3%20(40.8-52.2)%0747.6%20(44.3-51.1)%07%07%07%07AA%0737%20(5.1)%07107%20(14.8)%0746.6%20(40.2-53.1)%0738.9%20(35.1-42.7)%0743.9%20(35.7-52.9)%0743.7%20(38.8-49.0)%07%07%07%07Ptrend%07%07%070.66%070.22%070.25%070.39%07%07%07%07P%20i%07%07%070.33%070.55%07%07COMT%07rs4680%07GG%0744%20(6.1)%07143%20(19.7)%0743.1%20(37.1-49.1)%0742.0%20(38.7-45.3)%0743.3%20(35.7-51.7)%0748.8%20(44.3-53.6)%07%07%07%07GA%0796%20(13.2)%07275%20(37.9)%0745.3%20(41.3-49.3)%0743.3%20(41.0-45.7)%0746.4%20(41.1-52.1)%0746.7%20(43.5-50.1)%07%07%07%07AA%0734%20(4.7)%07133%20(18.3)%0743.4%20(36.7-50.0)%0738.6%20(35.2-42.0)%0752.6%20(43.3-62.9)%0743.6%20(39.3-48.2)%07%07%07%07Ptrend%07%07%070.93%070.17%070.15%070.12%07%07%07%07P%20i%07%07%070.47%070.05%07%07CYP1B1%07rs1056836%07CC%0753%20(7.3)%07163%20(22.4)%0746.6%20(41.1-52.1)%0742.8%20(39.7-45.8)%0754.2%20(46.4-62.7)%0747.2%20(43.1-51.5)%07%07%07%07CG%0786%20(11.8)%07282%20(38.8)%0746.3%20(42.0-50.5)%0741.8%20(39.5-44.1)%0747.0%20(41.3-53.0)%0746.4%20(43.3-49.6)%07%07%07%07GG%0735%20(4.8)%07108%20(14.9)%0736.9%20(30.3-43.5)%0740.6%20(36.8-44.3)%0737.0%20(29.5-45.4)%0745.5%20(40.6-50.7)%07%07%07%07Ptrend%07%07%070.04%070.38%070.004%070.62%07%07%07%07P%20i%07%07%070.19%070.02%07%07a%20Analyses%20are%20adjusted%20for%20age%20at%20mammography,%20body%20mass%20index,%20waist-to-hip%20ratio,%20height,%20age%20at%20menarche,%20age%20at%20first%20birth,%20number%20of%20full-term%20pregnancies,%20breastfeeding,%20number%20of%20breast%20biopsies,%20family%20history%20of%20breast%20cancer,%20past%20contraceptive%20and%20hormone%20replacement%20therapy%20uses,%20smoking%20status,%20energy%20and%20alcohol%20intakes,%20physical%20activity%20and%20education%20when%20applicable.%20Except%20for%20modifying%20effect%20of%20parity,%20models%20were%20also%20adjusted%20for%20age%20at%20first%20birth,%20number%20of%20full-term%20pregnancies%20and%20breastfeeding.%0Db%20%20SNPs:%20single%20nucleotide%20polymorphisms.%20They%20are%20identified%20by%20their%20dbSNP%20accession%20number%20at%20http://www.ncbi.nlm.nih.gov/SNP/.%0Dc%20Means%20of%20absolute%20density%20are%20presented%20as%20back-transformed%20values.%0Dd%20%20P%20value%20is%20the%20p%20trend,%20testing%20genotype%20dosage,%20number%20of%20copies%20of%20the%20rare%20allele%20entered%20as%200,%201,%202%20and%20mammographic%20density%20entered%20as%20a%20continuous%20variable.%0De%20%20P%20value%20for%20interaction%20between%20the%20variable%20(parity,%20hormonal%20derivative%20used,%20age%20at%20menarche%20or%20body%20mass%20index)%20and%20the%20genotype%20dosage%20%0Dfrom%20linear%20regression.%07%07) |  |  | **No** | | **Yes** | **No** | **Yes** | **No** | **Yes** |
| [ERα](../%0DAdditional%20file%201.%20Modifying%20effect%20of%20parity%20on%20the%20association%20between%20SNPs%20and%20mammographic%20density%07%07Gene%07SNP%07%07%07Adjusted%20mean%20mammographic%20densitya%07%07Name%07Reference%20IDb%07Genotype%07N(%25)%07Percent%20density%20(%25)%07Absolute%20density%20(cm2)c%07%07Parity%07%07%07No%07Yes%07No%07Yes%07No%07Yes%07%07ERα%07rs2077647%07TT%0751%20(7.1)%07137%20(19.1)%0744.6%20(39.2-50.1)%0741.8%20(38.4-45.1)%0750.3%20(42.8-58.5)%0746.6%20(42.1-51.4)%07%07%07%07TC%0782%20(11.4)%07270%20(37.6)%0744.6%20(40.3-49.0)%0741.9%20(39.6-44.3)%0746.5%20(40.7-52.7)%0747.4%20(44.2-50.8)%07%07%07%07CC%0739%20(5.4)%07139%20(19.4)%0745.2%20(38.8-51.6)%0742.7%20(39.4-46.0)%0743.4%20(35.4-52.3)%0745.0%20(40.7-49.6)%07%07%07%07Ptrendd%07%07%070.91%070.70%070.24%070.62%07%07%07%07P%20ie%07%07%070.93%070.43%07%07%07rs2234693%07AA%0752%20(7.2)%07157%20(21.7)%0744.0%20(38.5-49.4)%0741.7%20(38.5-44.8)%0750.9%20(43.4-59.1)%0747.6%20(43.3-52.0)%07%07%07%07AG%0787%20(12.0)%07259%20(35.7)%0745.7%20(41.4-49.9)%0741.1%20(38.7-43.5)%0746.6%20(41.0-52.7)%0746.9%20(43.6-50.3)%07%07%07%07GG%0734%20(4.7)%07136%20(18.8)%0744.6%20(37.8-51.4)%0743.3%20(39.9-46.6)%0744.6%20(36.0-54.2)%0744.2%20(39.8-48.8)%07%07%07%07Ptrend%07%07%070.82%070.51%070.28%070.30%07%07%07%07P%20i%07%07%070.91%070.65%07%07%07rs9340799%07AA%0781%20(11.2)%07218%20(30.1)%0744.4%20(40.1-48.8)%0742.2%20(39.6-44.8)%0750.1%20(44.1-56.6)%0749.2%20(45.5-53.0)%07%07%07%07AG%0769%20(9.5)%07252%20(34.8)%0745.0%20(40.2-49.7)%0740.9%20(38.4-43.3)%0743.5%20(37.5-50.1)%0744.9%20(41.7-48.3)%07%07%07%07GG%0723%20(3.2)%0782%20(11.3)%0747.3%20(39.1-55.6)%0744.0%20(39.7-48.3)%0751.3%20(40.1-64.0)%0744.7%20(39.2-50.7)%07%07%07%07Ptrend%07%07%070.58%070.76%070.65%070.11%07%07%07%07P%20i%07%07%070.74%070.70%07%07%07rs2228480%07CC%07126%20(17.4)%07376%20(51.8)%0744.8%20(41.3-48.3)%0741.1%20(39.1-43.1)%0748.1%20(43.4-53.2)%0745.5%20(42.8-48.2)%07%07%07%07CT%0746%20(6.3)%07157%20(21.6)%0745.2%20(39.4-51.0)%0743.0%20(39.9-46.2)%0744.5%20(37.1-52.7)%0748.1%20(43.9-52.6)%07%07%07%07TT%072%20(0.3)%0719%20(2.6)%0716.9%20(-10.7-44.4)%0748.9%20(39.9-57.8)%0729.2%20(8.6-66.9)%0757.3%20(44.4-72.0)%07%07%07%07Ptrend%07%07%070.51%070.09%070.27%070.08%07%07%07%07P%20i%07%07%070.18%070.08%07%07ERβ%07rs3829768%07TT%07173%20(23.9)%07550%20(75.9)%0744.8%20(41.8-47.8)%0741.9%20(40.2-43.5)%0747.2%20(43.1-51.5)%0746.5%20(44.3-48.8)%07%07%07%07TC%071%20(0.1)%071%20(0.1)%07-8.2%20(-47.1-30.7)%0741.6%20(2.4-80.8)%076.0%20(12.3-33.8)%0792.5%20(31.7-187.9)%07%07%07%07Ptrend%07%07%070.008%070.99%070.01%070.17%07%07%07%07P%20i%07%07%070.06%070.006%07%07%07rs1256049%07GG%07162%20(22.3)%07517%20(71.1)%0745.4%20(42.3-48.5)%0741.9%20(40.2-43.6)%0748.0%20(43.7-52.4)%0746.6%20(44.2-49.0)%07%07%07%07GA%0712%20(1.7)%0736%20(5.0)%0737.9%20(26.6-49.2)%0740.8%20(34.3-47.4)%0741.0%20(27.9-56.9)%0746.1%20(37.6-55.6)%07%07%07%07AA%070%070%07%07%07%07%07%07%07%07Ptrend%07%07%070.21%070.76%070.38%070.92%07%07%07%07P%20i%07%07%070.35%070.48%07%07HSD17B1%07rs676387%07GG%0788%20(12.1)%07296%20(40.7)%0746.0%20(41.8-50.2)%0740.4%20(38.2-42.7)%0745.1%20(39.6-51.0)%0745.2%20(42.2-48.4)%07%07%07%07GT%0775%20(10.3)%07212%20(29.2)%0742.8%20(38.3-47.4)%0743.8%20(41.1-46.4)%0748.7%20(42.5-55.2)%0748.4%20(44.7-52.2)%07%07%07%07TT%0711%20(1.5)%0745%20(6.2)%0743.4%20(31.7-55.2)%0742.1%20(36.3-47.9)%0748.8%20(33.8-67.0)%0746.6%20(39.0-55.0)%07%07%07%07Ptrend%07%07%070.37%070.16%070.42%070.34%07%07%07%07P%20i%07%07%070.14%070.80%07%07%07rs598126%07CC%0742%20(5.8)%07149%20(20.6)%0744.5%20(38.4-50.5)%0742.2%20(39.0-45.4)%0751.1%20(42.8-60.3)%0748.1%20(43.8-52.7)%07%07%07%07CT%0787%20(12.1)%07263%20(36.4)%0743.5%20(39.3-47.7)%0743.0%20(40.6-45.4)%0745.9%20(40.4-51.9)%0746.8%20(43.5-50.1)%07%07%07%07TT%0744%20(6.1)%07137%20(19.0)%0746.8%20(40.9-52.7)%0738.9%20(35.5-42.3)%0745.0%20(37.4-53.4)%0743.8%20(39.4-48.4)%07%07%07%07Ptrend%07%07%070.58%070.18%070.31%070.18%07%07%07%07P%20i%07%07%070.26%070.81%07%07%07rs2010750%07GG%0747%20(6.5)%07186%20(25.7)%0744.5%20(38.8-50.2)%0742.4%20(39.5-45.2)%0750.8%20(42.9-59.4)%0747.1%20(43.2-51.1)%07%07%07%07GA%0789%20(12.3)%07258%20(35.6)%0743.9%20(39.7-48.1)%0742.8%20(40.4-45.2)%0746.3%20(40.8-52.2)%0747.6%20(44.3-51.1)%07%07%07%07AA%0737%20(5.1)%07107%20(14.8)%0746.6%20(40.2-53.1)%0738.9%20(35.1-42.7)%0743.9%20(35.7-52.9)%0743.7%20(38.8-49.0)%07%07%07%07Ptrend%07%07%070.66%070.22%070.25%070.39%07%07%07%07P%20i%07%07%070.33%070.55%07%07COMT%07rs4680%07GG%0744%20(6.1)%07143%20(19.7)%0743.1%20(37.1-49.1)%0742.0%20(38.7-45.3)%0743.3%20(35.7-51.7)%0748.8%20(44.3-53.6)%07%07%07%07GA%0796%20(13.2)%07275%20(37.9)%0745.3%20(41.3-49.3)%0743.3%20(41.0-45.7)%0746.4%20(41.1-52.1)%0746.7%20(43.5-50.1)%07%07%07%07AA%0734%20(4.7)%07133%20(18.3)%0743.4%20(36.7-50.0)%0738.6%20(35.2-42.0)%0752.6%20(43.3-62.9)%0743.6%20(39.3-48.2)%07%07%07%07Ptrend%07%07%070.93%070.17%070.15%070.12%07%07%07%07P%20i%07%07%070.47%070.05%07%07CYP1B1%07rs1056836%07CC%0753%20(7.3)%07163%20(22.4)%0746.6%20(41.1-52.1)%0742.8%20(39.7-45.8)%0754.2%20(46.4-62.7)%0747.2%20(43.1-51.5)%07%07%07%07CG%0786%20(11.8)%07282%20(38.8)%0746.3%20(42.0-50.5)%0741.8%20(39.5-44.1)%0747.0%20(41.3-53.0)%0746.4%20(43.3-49.6)%07%07%07%07GG%0735%20(4.8)%07108%20(14.9)%0736.9%20(30.3-43.5)%0740.6%20(36.8-44.3)%0737.0%20(29.5-45.4)%0745.5%20(40.6-50.7)%07%07%07%07Ptrend%07%07%070.04%070.38%070.004%070.62%07%07%07%07P%20i%07%07%070.19%070.02%07%07a%20Analyses%20are%20adjusted%20for%20age%20at%20mammography,%20body%20mass%20index,%20waist-to-hip%20ratio,%20height,%20age%20at%20menarche,%20age%20at%20first%20birth,%20number%20of%20full-term%20pregnancies,%20breastfeeding,%20number%20of%20breast%20biopsies,%20family%20history%20of%20breast%20cancer,%20past%20contraceptive%20and%20hormone%20replacement%20therapy%20uses,%20smoking%20status,%20energy%20and%20alcohol%20intakes,%20physical%20activity%20and%20education%20when%20applicable.%20Except%20for%20modifying%20effect%20of%20parity,%20models%20were%20also%20adjusted%20for%20age%20at%20first%20birth,%20number%20of%20full-term%20pregnancies%20and%20breastfeeding.%0Db%20%20SNPs:%20single%20nucleotide%20polymorphisms.%20They%20are%20identified%20by%20their%20dbSNP%20accession%20number%20at%20http://www.ncbi.nlm.nih.gov/SNP/.%0Dc%20Means%20of%20absolute%20density%20are%20presented%20as%20back-transformed%20values.%0Dd%20%20P%20value%20is%20the%20p%20trend,%20testing%20genotype%20dosage,%20number%20of%20copies%20of%20the%20rare%20allele%20entered%20as%200,%201,%202%20and%20mammographic%20density%20entered%20as%20a%20continuous%20variable.%0De%20%20P%20value%20for%20interaction%20between%20the%20variable%20(parity,%20hormonal%20derivative%20used,%20age%20at%20menarche%20or%20body%20mass%20index)%20and%20the%20genotype%20dosage%20%0Dfrom%20linear%20regression.%07%07) | rs2077647 | TT | 51 (7.1) | 137 (19.1) | | 44.6 (39.2-50.1) | 41.8 (38.4-45.1) | 50.3 (42.8-58.5) | 46.6 (42.1-51.4) |
|  |  | TC | 82 (11.4) | 270 (37.6) | | 44.6 (40.3-49.0) | 41.9 (39.6-44.3) | 46.5 (40.7-52.7) | 47.4 (44.2-50.8) |
|  |  | CC | 39 (5.4) | 139 (19.4) | | 45.2 (38.8-51.6) | 42.7 (39.4-46.0) | 43.4 (35.4-52.3) | 45.0 (40.7-49.6) |
|  |  | *P*_trend_^d^ |  |  | | 0.91 | 0.70 | 0.24 | 0.62 |
|  |  | *P* _i_^e^ |  |  | | 0.93 | | 0.43 | |
|  | rs2234693 | AA | 52 (7.2) | 157 (21.7) | | 44.0 (38.5-49.4) | 41.7 (38.5-44.8) | 50.9 (43.4-59.1) | 47.6 (43.3-52.0) |
|  |  | AG | 87 (12.0) | 259 (35.7) | | 45.7 (41.4-49.9) | 41.1 (38.7-43.5) | 46.6 (41.0-52.7) | 46.9 (43.6-50.3) |
|  |  | GG | 34 (4.7) | 136 (18.8) | | 44.6 (37.8-51.4) | 43.3 (39.9-46.6) | 44.6 (36.0-54.2) | 44.2 (39.8-48.8) |
|  |  | *P*_trend_ |  |  | | 0.82 | 0.51 | 0.28 | 0.30 |
|  |  | *P* _i_ |  |  | | 0.91 | | 0.65 | |
|  | rs9340799 | AA | 81 (11.2) | 218 (30.1) | | 44.4 (40.1-48.8) | 42.2 (39.6-44.8) | 50.1 (44.1-56.6) | 49.2 (45.5-53.0) |
|  |  | AG | 69 (9.5) | 252 (34.8) | | 45.0 (40.2-49.7) | 40.9 (38.4-43.3) | 43.5 (37.5-50.1) | 44.9 (41.7-48.3) |
|  |  | GG | 23 (3.2) | 82 (11.3) | | 47.3 (39.1-55.6) | 44.0 (39.7-48.3) | 51.3 (40.1-64.0) | 44.7 (39.2-50.7) |
|  |  | *P*_trend_ |  |  | | 0.58 | 0.76 | 0.65 | 0.11 |
|  |  | *P* _i_ |  |  | | 0.74 | | 0.70 | |
|  | rs2228480 | CC | 126 (17.4) | 376 (51.8) | | 44.8 (41.3-48.3) | 41.1 (39.1-43.1) | 48.1 (43.4-53.2) | 45.5 (42.8-48.2) |
|  |  | CT | 46 (6.3) | 157 (21.6) | | 45.2 (39.4-51.0) | 43.0 (39.9-46.2) | 44.5 (37.1-52.7) | 48.1 (43.9-52.6) |
|  |  | TT | 2 (0.3) | 19 (2.6) | | 16.9 (-10.7-44.4) | 48.9 (39.9-57.8) | 29.2 (8.6-66.9) | 57.3 (44.4-72.0) |
|  |  | *P*_trend_ |  |  | | 0.51 | 0.09 | 0.27 | 0.08 |
|  |  | *P* _i_ |  |  | | 0.18 | | 0.08 | |
| [ERβ](../%0DAdditional%20file%201.%20Modifying%20effect%20of%20parity%20on%20the%20association%20between%20SNPs%20and%20mammographic%20density%07%07Gene%07SNP%07%07%07Adjusted%20mean%20mammographic%20densitya%07%07Name%07Reference%20IDb%07Genotype%07N(%25)%07Percent%20density%20(%25)%07Absolute%20density%20(cm2)c%07%07Parity%07%07%07No%07Yes%07No%07Yes%07No%07Yes%07%07ERα%07rs2077647%07TT%0751%20(7.1)%07137%20(19.1)%0744.6%20(39.2-50.1)%0741.8%20(38.4-45.1)%0750.3%20(42.8-58.5)%0746.6%20(42.1-51.4)%07%07%07%07TC%0782%20(11.4)%07270%20(37.6)%0744.6%20(40.3-49.0)%0741.9%20(39.6-44.3)%0746.5%20(40.7-52.7)%0747.4%20(44.2-50.8)%07%07%07%07CC%0739%20(5.4)%07139%20(19.4)%0745.2%20(38.8-51.6)%0742.7%20(39.4-46.0)%0743.4%20(35.4-52.3)%0745.0%20(40.7-49.6)%07%07%07%07Ptrendd%07%07%070.91%070.70%070.24%070.62%07%07%07%07P%20ie%07%07%070.93%070.43%07%07%07rs2234693%07AA%0752%20(7.2)%07157%20(21.7)%0744.0%20(38.5-49.4)%0741.7%20(38.5-44.8)%0750.9%20(43.4-59.1)%0747.6%20(43.3-52.0)%07%07%07%07AG%0787%20(12.0)%07259%20(35.7)%0745.7%20(41.4-49.9)%0741.1%20(38.7-43.5)%0746.6%20(41.0-52.7)%0746.9%20(43.6-50.3)%07%07%07%07GG%0734%20(4.7)%07136%20(18.8)%0744.6%20(37.8-51.4)%0743.3%20(39.9-46.6)%0744.6%20(36.0-54.2)%0744.2%20(39.8-48.8)%07%07%07%07Ptrend%07%07%070.82%070.51%070.28%070.30%07%07%07%07P%20i%07%07%070.91%070.65%07%07%07rs9340799%07AA%0781%20(11.2)%07218%20(30.1)%0744.4%20(40.1-48.8)%0742.2%20(39.6-44.8)%0750.1%20(44.1-56.6)%0749.2%20(45.5-53.0)%07%07%07%07AG%0769%20(9.5)%07252%20(34.8)%0745.0%20(40.2-49.7)%0740.9%20(38.4-43.3)%0743.5%20(37.5-50.1)%0744.9%20(41.7-48.3)%07%07%07%07GG%0723%20(3.2)%0782%20(11.3)%0747.3%20(39.1-55.6)%0744.0%20(39.7-48.3)%0751.3%20(40.1-64.0)%0744.7%20(39.2-50.7)%07%07%07%07Ptrend%07%07%070.58%070.76%070.65%070.11%07%07%07%07P%20i%07%07%070.74%070.70%07%07%07rs2228480%07CC%07126%20(17.4)%07376%20(51.8)%0744.8%20(41.3-48.3)%0741.1%20(39.1-43.1)%0748.1%20(43.4-53.2)%0745.5%20(42.8-48.2)%07%07%07%07CT%0746%20(6.3)%07157%20(21.6)%0745.2%20(39.4-51.0)%0743.0%20(39.9-46.2)%0744.5%20(37.1-52.7)%0748.1%20(43.9-52.6)%07%07%07%07TT%072%20(0.3)%0719%20(2.6)%0716.9%20(-10.7-44.4)%0748.9%20(39.9-57.8)%0729.2%20(8.6-66.9)%0757.3%20(44.4-72.0)%07%07%07%07Ptrend%07%07%070.51%070.09%070.27%070.08%07%07%07%07P%20i%07%07%070.18%070.08%07%07ERβ%07rs3829768%07TT%07173%20(23.9)%07550%20(75.9)%0744.8%20(41.8-47.8)%0741.9%20(40.2-43.5)%0747.2%20(43.1-51.5)%0746.5%20(44.3-48.8)%07%07%07%07TC%071%20(0.1)%071%20(0.1)%07-8.2%20(-47.1-30.7)%0741.6%20(2.4-80.8)%076.0%20(12.3-33.8)%0792.5%20(31.7-187.9)%07%07%07%07Ptrend%07%07%070.008%070.99%070.01%070.17%07%07%07%07P%20i%07%07%070.06%070.006%07%07%07rs1256049%07GG%07162%20(22.3)%07517%20(71.1)%0745.4%20(42.3-48.5)%0741.9%20(40.2-43.6)%0748.0%20(43.7-52.4)%0746.6%20(44.2-49.0)%07%07%07%07GA%0712%20(1.7)%0736%20(5.0)%0737.9%20(26.6-49.2)%0740.8%20(34.3-47.4)%0741.0%20(27.9-56.9)%0746.1%20(37.6-55.6)%07%07%07%07AA%070%070%07%07%07%07%07%07%07%07Ptrend%07%07%070.21%070.76%070.38%070.92%07%07%07%07P%20i%07%07%070.35%070.48%07%07HSD17B1%07rs676387%07GG%0788%20(12.1)%07296%20(40.7)%0746.0%20(41.8-50.2)%0740.4%20(38.2-42.7)%0745.1%20(39.6-51.0)%0745.2%20(42.2-48.4)%07%07%07%07GT%0775%20(10.3)%07212%20(29.2)%0742.8%20(38.3-47.4)%0743.8%20(41.1-46.4)%0748.7%20(42.5-55.2)%0748.4%20(44.7-52.2)%07%07%07%07TT%0711%20(1.5)%0745%20(6.2)%0743.4%20(31.7-55.2)%0742.1%20(36.3-47.9)%0748.8%20(33.8-67.0)%0746.6%20(39.0-55.0)%07%07%07%07Ptrend%07%07%070.37%070.16%070.42%070.34%07%07%07%07P%20i%07%07%070.14%070.80%07%07%07rs598126%07CC%0742%20(5.8)%07149%20(20.6)%0744.5%20(38.4-50.5)%0742.2%20(39.0-45.4)%0751.1%20(42.8-60.3)%0748.1%20(43.8-52.7)%07%07%07%07CT%0787%20(12.1)%07263%20(36.4)%0743.5%20(39.3-47.7)%0743.0%20(40.6-45.4)%0745.9%20(40.4-51.9)%0746.8%20(43.5-50.1)%07%07%07%07TT%0744%20(6.1)%07137%20(19.0)%0746.8%20(40.9-52.7)%0738.9%20(35.5-42.3)%0745.0%20(37.4-53.4)%0743.8%20(39.4-48.4)%07%07%07%07Ptrend%07%07%070.58%070.18%070.31%070.18%07%07%07%07P%20i%07%07%070.26%070.81%07%07%07rs2010750%07GG%0747%20(6.5)%07186%20(25.7)%0744.5%20(38.8-50.2)%0742.4%20(39.5-45.2)%0750.8%20(42.9-59.4)%0747.1%20(43.2-51.1)%07%07%07%07GA%0789%20(12.3)%07258%20(35.6)%0743.9%20(39.7-48.1)%0742.8%20(40.4-45.2)%0746.3%20(40.8-52.2)%0747.6%20(44.3-51.1)%07%07%07%07AA%0737%20(5.1)%07107%20(14.8)%0746.6%20(40.2-53.1)%0738.9%20(35.1-42.7)%0743.9%20(35.7-52.9)%0743.7%20(38.8-49.0)%07%07%07%07Ptrend%07%07%070.66%070.22%070.25%070.39%07%07%07%07P%20i%07%07%070.33%070.55%07%07COMT%07rs4680%07GG%0744%20(6.1)%07143%20(19.7)%0743.1%20(37.1-49.1)%0742.0%20(38.7-45.3)%0743.3%20(35.7-51.7)%0748.8%20(44.3-53.6)%07%07%07%07GA%0796%20(13.2)%07275%20(37.9)%0745.3%20(41.3-49.3)%0743.3%20(41.0-45.7)%0746.4%20(41.1-52.1)%0746.7%20(43.5-50.1)%07%07%07%07AA%0734%20(4.7)%07133%20(18.3)%0743.4%20(36.7-50.0)%0738.6%20(35.2-42.0)%0752.6%20(43.3-62.9)%0743.6%20(39.3-48.2)%07%07%07%07Ptrend%07%07%070.93%070.17%070.15%070.12%07%07%07%07P%20i%07%07%070.47%070.05%07%07CYP1B1%07rs1056836%07CC%0753%20(7.3)%07163%20(22.4)%0746.6%20(41.1-52.1)%0742.8%20(39.7-45.8)%0754.2%20(46.4-62.7)%0747.2%20(43.1-51.5)%07%07%07%07CG%0786%20(11.8)%07282%20(38.8)%0746.3%20(42.0-50.5)%0741.8%20(39.5-44.1)%0747.0%20(41.3-53.0)%0746.4%20(43.3-49.6)%07%07%07%07GG%0735%20(4.8)%07108%20(14.9)%0736.9%20(30.3-43.5)%0740.6%20(36.8-44.3)%0737.0%20(29.5-45.4)%0745.5%20(40.6-50.7)%07%07%07%07Ptrend%07%07%070.04%070.38%070.004%070.62%07%07%07%07P%20i%07%07%070.19%070.02%07%07a%20Analyses%20are%20adjusted%20for%20age%20at%20mammography,%20body%20mass%20index,%20waist-to-hip%20ratio,%20height,%20age%20at%20menarche,%20age%20at%20first%20birth,%20number%20of%20full-term%20pregnancies,%20breastfeeding,%20number%20of%20breast%20biopsies,%20family%20history%20of%20breast%20cancer,%20past%20contraceptive%20and%20hormone%20replacement%20therapy%20uses,%20smoking%20status,%20energy%20and%20alcohol%20intakes,%20physical%20activity%20and%20education%20when%20applicable.%20Except%20for%20modifying%20effect%20of%20parity,%20models%20were%20also%20adjusted%20for%20age%20at%20first%20birth,%20number%20of%20full-term%20pregnancies%20and%20breastfeeding.%0Db%20%20SNPs:%20single%20nucleotide%20polymorphisms.%20They%20are%20identified%20by%20their%20dbSNP%20accession%20number%20at%20http://www.ncbi.nlm.nih.gov/SNP/.%0Dc%20Means%20of%20absolute%20density%20are%20presented%20as%20back-transformed%20values.%0Dd%20%20P%20value%20is%20the%20p%20trend,%20testing%20genotype%20dosage,%20number%20of%20copies%20of%20the%20rare%20allele%20entered%20as%200,%201,%202%20and%20mammographic%20density%20entered%20as%20a%20continuous%20variable.%0De%20%20P%20value%20for%20interaction%20between%20the%20variable%20(parity,%20hormonal%20derivative%20used,%20age%20at%20menarche%20or%20body%20mass%20index)%20and%20the%20genotype%20dosage%20%0Dfrom%20linear%20regression.%07%07) | rs3829768 | TT | 173 (23.9) | 550 (75.9) | | N/A^f^ | N/A | N/A | N/A |
|  |  | TC | 1 (0.1) | 1 (0.1) | | N/A | N/A | N/A | N/A |
|  |  | *P*_trend_ |  |  | | N/A | N/A | N/A | N/A |
|  |  | *P* _i_ |  |  | | N/A | | N/A | |
|  | rs1256049 | GG | 162 (22.3) | 517 (71.1) | | 45.4 (42.3-48.5) | 41.9 (40.2-43.6) | 48.0 (43.7-52.4) | 46.6 (44.2-49.0) |
|  |  | GA | 12 (1.7) | 36 (5.0) | | 37.9 (26.6-49.2) | 40.8 (34.3-47.4) | 41.0 (27.9-56.9) | 46.1 (37.6-55.6) |
|  |  | *P*_trend_ |  |  | | 0.21 | 0.76 | 0.38 | 0.92 |
|  |  | *P* _i_ |  |  | | 0.35 | | 0.48 | |
| [HSD17B1](../%0DAdditional%20file%201.%20Modifying%20effect%20of%20parity%20on%20the%20association%20between%20SNPs%20and%20mammographic%20density%07%07Gene%07SNP%07%07%07Adjusted%20mean%20mammographic%20densitya%07%07Name%07Reference%20IDb%07Genotype%07N(%25)%07Percent%20density%20(%25)%07Absolute%20density%20(cm2)c%07%07Parity%07%07%07No%07Yes%07No%07Yes%07No%07Yes%07%07ERα%07rs2077647%07TT%0751%20(7.1)%07137%20(19.1)%0744.6%20(39.2-50.1)%0741.8%20(38.4-45.1)%0750.3%20(42.8-58.5)%0746.6%20(42.1-51.4)%07%07%07%07TC%0782%20(11.4)%07270%20(37.6)%0744.6%20(40.3-49.0)%0741.9%20(39.6-44.3)%0746.5%20(40.7-52.7)%0747.4%20(44.2-50.8)%07%07%07%07CC%0739%20(5.4)%07139%20(19.4)%0745.2%20(38.8-51.6)%0742.7%20(39.4-46.0)%0743.4%20(35.4-52.3)%0745.0%20(40.7-49.6)%07%07%07%07Ptrendd%07%07%070.91%070.70%070.24%070.62%07%07%07%07P%20ie%07%07%070.93%070.43%07%07%07rs2234693%07AA%0752%20(7.2)%07157%20(21.7)%0744.0%20(38.5-49.4)%0741.7%20(38.5-44.8)%0750.9%20(43.4-59.1)%0747.6%20(43.3-52.0)%07%07%07%07AG%0787%20(12.0)%07259%20(35.7)%0745.7%20(41.4-49.9)%0741.1%20(38.7-43.5)%0746.6%20(41.0-52.7)%0746.9%20(43.6-50.3)%07%07%07%07GG%0734%20(4.7)%07136%20(18.8)%0744.6%20(37.8-51.4)%0743.3%20(39.9-46.6)%0744.6%20(36.0-54.2)%0744.2%20(39.8-48.8)%07%07%07%07Ptrend%07%07%070.82%070.51%070.28%070.30%07%07%07%07P%20i%07%07%070.91%070.65%07%07%07rs9340799%07AA%0781%20(11.2)%07218%20(30.1)%0744.4%20(40.1-48.8)%0742.2%20(39.6-44.8)%0750.1%20(44.1-56.6)%0749.2%20(45.5-53.0)%07%07%07%07AG%0769%20(9.5)%07252%20(34.8)%0745.0%20(40.2-49.7)%0740.9%20(38.4-43.3)%0743.5%20(37.5-50.1)%0744.9%20(41.7-48.3)%07%07%07%07GG%0723%20(3.2)%0782%20(11.3)%0747.3%20(39.1-55.6)%0744.0%20(39.7-48.3)%0751.3%20(40.1-64.0)%0744.7%20(39.2-50.7)%07%07%07%07Ptrend%07%07%070.58%070.76%070.65%070.11%07%07%07%07P%20i%07%07%070.74%070.70%07%07%07rs2228480%07CC%07126%20(17.4)%07376%20(51.8)%0744.8%20(41.3-48.3)%0741.1%20(39.1-43.1)%0748.1%20(43.4-53.2)%0745.5%20(42.8-48.2)%07%07%07%07CT%0746%20(6.3)%07157%20(21.6)%0745.2%20(39.4-51.0)%0743.0%20(39.9-46.2)%0744.5%20(37.1-52.7)%0748.1%20(43.9-52.6)%07%07%07%07TT%072%20(0.3)%0719%20(2.6)%0716.9%20(-10.7-44.4)%0748.9%20(39.9-57.8)%0729.2%20(8.6-66.9)%0757.3%20(44.4-72.0)%07%07%07%07Ptrend%07%07%070.51%070.09%070.27%070.08%07%07%07%07P%20i%07%07%070.18%070.08%07%07ERβ%07rs3829768%07TT%07173%20(23.9)%07550%20(75.9)%0744.8%20(41.8-47.8)%0741.9%20(40.2-43.5)%0747.2%20(43.1-51.5)%0746.5%20(44.3-48.8)%07%07%07%07TC%071%20(0.1)%071%20(0.1)%07-8.2%20(-47.1-30.7)%0741.6%20(2.4-80.8)%076.0%20(12.3-33.8)%0792.5%20(31.7-187.9)%07%07%07%07Ptrend%07%07%070.008%070.99%070.01%070.17%07%07%07%07P%20i%07%07%070.06%070.006%07%07%07rs1256049%07GG%07162%20(22.3)%07517%20(71.1)%0745.4%20(42.3-48.5)%0741.9%20(40.2-43.6)%0748.0%20(43.7-52.4)%0746.6%20(44.2-49.0)%07%07%07%07GA%0712%20(1.7)%0736%20(5.0)%0737.9%20(26.6-49.2)%0740.8%20(34.3-47.4)%0741.0%20(27.9-56.9)%0746.1%20(37.6-55.6)%07%07%07%07AA%070%070%07%07%07%07%07%07%07%07Ptrend%07%07%070.21%070.76%070.38%070.92%07%07%07%07P%20i%07%07%070.35%070.48%07%07HSD17B1%07rs676387%07GG%0788%20(12.1)%07296%20(40.7)%0746.0%20(41.8-50.2)%0740.4%20(38.2-42.7)%0745.1%20(39.6-51.0)%0745.2%20(42.2-48.4)%07%07%07%07GT%0775%20(10.3)%07212%20(29.2)%0742.8%20(38.3-47.4)%0743.8%20(41.1-46.4)%0748.7%20(42.5-55.2)%0748.4%20(44.7-52.2)%07%07%07%07TT%0711%20(1.5)%0745%20(6.2)%0743.4%20(31.7-55.2)%0742.1%20(36.3-47.9)%0748.8%20(33.8-67.0)%0746.6%20(39.0-55.0)%07%07%07%07Ptrend%07%07%070.37%070.16%070.42%070.34%07%07%07%07P%20i%07%07%070.14%070.80%07%07%07rs598126%07CC%0742%20(5.8)%07149%20(20.6)%0744.5%20(38.4-50.5)%0742.2%20(39.0-45.4)%0751.1%20(42.8-60.3)%0748.1%20(43.8-52.7)%07%07%07%07CT%0787%20(12.1)%07263%20(36.4)%0743.5%20(39.3-47.7)%0743.0%20(40.6-45.4)%0745.9%20(40.4-51.9)%0746.8%20(43.5-50.1)%07%07%07%07TT%0744%20(6.1)%07137%20(19.0)%0746.8%20(40.9-52.7)%0738.9%20(35.5-42.3)%0745.0%20(37.4-53.4)%0743.8%20(39.4-48.4)%07%07%07%07Ptrend%07%07%070.58%070.18%070.31%070.18%07%07%07%07P%20i%07%07%070.26%070.81%07%07%07rs2010750%07GG%0747%20(6.5)%07186%20(25.7)%0744.5%20(38.8-50.2)%0742.4%20(39.5-45.2)%0750.8%20(42.9-59.4)%0747.1%20(43.2-51.1)%07%07%07%07GA%0789%20(12.3)%07258%20(35.6)%0743.9%20(39.7-48.1)%0742.8%20(40.4-45.2)%0746.3%20(40.8-52.2)%0747.6%20(44.3-51.1)%07%07%07%07AA%0737%20(5.1)%07107%20(14.8)%0746.6%20(40.2-53.1)%0738.9%20(35.1-42.7)%0743.9%20(35.7-52.9)%0743.7%20(38.8-49.0)%07%07%07%07Ptrend%07%07%070.66%070.22%070.25%070.39%07%07%07%07P%20i%07%07%070.33%070.55%07%07COMT%07rs4680%07GG%0744%20(6.1)%07143%20(19.7)%0743.1%20(37.1-49.1)%0742.0%20(38.7-45.3)%0743.3%20(35.7-51.7)%0748.8%20(44.3-53.6)%07%07%07%07GA%0796%20(13.2)%07275%20(37.9)%0745.3%20(41.3-49.3)%0743.3%20(41.0-45.7)%0746.4%20(41.1-52.1)%0746.7%20(43.5-50.1)%07%07%07%07AA%0734%20(4.7)%07133%20(18.3)%0743.4%20(36.7-50.0)%0738.6%20(35.2-42.0)%0752.6%20(43.3-62.9)%0743.6%20(39.3-48.2)%07%07%07%07Ptrend%07%07%070.93%070.17%070.15%070.12%07%07%07%07P%20i%07%07%070.47%070.05%07%07CYP1B1%07rs1056836%07CC%0753%20(7.3)%07163%20(22.4)%0746.6%20(41.1-52.1)%0742.8%20(39.7-45.8)%0754.2%20(46.4-62.7)%0747.2%20(43.1-51.5)%07%07%07%07CG%0786%20(11.8)%07282%20(38.8)%0746.3%20(42.0-50.5)%0741.8%20(39.5-44.1)%0747.0%20(41.3-53.0)%0746.4%20(43.3-49.6)%07%07%07%07GG%0735%20(4.8)%07108%20(14.9)%0736.9%20(30.3-43.5)%0740.6%20(36.8-44.3)%0737.0%20(29.5-45.4)%0745.5%20(40.6-50.7)%07%07%07%07Ptrend%07%07%070.04%070.38%070.004%070.62%07%07%07%07P%20i%07%07%070.19%070.02%07%07a%20Analyses%20are%20adjusted%20for%20age%20at%20mammography,%20body%20mass%20index,%20waist-to-hip%20ratio,%20height,%20age%20at%20menarche,%20age%20at%20first%20birth,%20number%20of%20full-term%20pregnancies,%20breastfeeding,%20number%20of%20breast%20biopsies,%20family%20history%20of%20breast%20cancer,%20past%20contraceptive%20and%20hormone%20replacement%20therapy%20uses,%20smoking%20status,%20energy%20and%20alcohol%20intakes,%20physical%20activity%20and%20education%20when%20applicable.%20Except%20for%20modifying%20effect%20of%20parity,%20models%20were%20also%20adjusted%20for%20age%20at%20first%20birth,%20number%20of%20full-term%20pregnancies%20and%20breastfeeding.%0Db%20%20SNPs:%20single%20nucleotide%20polymorphisms.%20They%20are%20identified%20by%20their%20dbSNP%20accession%20number%20at%20http://www.ncbi.nlm.nih.gov/SNP/.%0Dc%20Means%20of%20absolute%20density%20are%20presented%20as%20back-transformed%20values.%0Dd%20%20P%20value%20is%20the%20p%20trend,%20testing%20genotype%20dosage,%20number%20of%20copies%20of%20the%20rare%20allele%20entered%20as%200,%201,%202%20and%20mammographic%20density%20entered%20as%20a%20continuous%20variable.%0De%20%20P%20value%20for%20interaction%20between%20the%20variable%20(parity,%20hormonal%20derivative%20used,%20age%20at%20menarche%20or%20body%20mass%20index)%20and%20the%20genotype%20dosage%20%0Dfrom%20linear%20regression.%07%07) | rs676387 | GG | 88 (12.1) | 296 (40.7) | | 46.0 (41.8-50.2) | 40.4 (38.2-42.7) | 45.1 (39.6-51.0) | 45.2 (42.2-48.4) |
|  |  | GT | 75 (10.3) | 212 (29.2) | | 42.8 (38.3-47.4) | 43.8 (41.1-46.4) | 48.7 (42.5-55.2) | 48.4 (44.7-52.2) |
|  |  | TT | 11 (1.5) | 45 (6.2) | | 43.4 (31.7-55.2) | 42.1 (36.3-47.9) | 48.8 (33.8-67.0) | 46.6 (39.0-55.0) |
|  |  | *P*_trend_ |  |  | | 0.37 | 0.16 | 0.42 | 0.34 |
|  |  | *P* _i_ |  |  | | 0.14 | | 0.80 | |
|  | rs598126 | CC | 42 (5.8) | 149 (20.6) | | 44.5 (38.4-50.5) | 42.2 (39.0-45.4) | 51.1 (42.8-60.3) | 48.1 (43.8-52.7) |
|  |  | CT | 87 (12.1) | 263 (36.4) | | 43.5 (39.3-47.7) | 43.0 (40.6-45.4) | 45.9 (40.4-51.9) | 46.8 (43.5-50.1) |
|  |  | TT | 44 (6.1) | 137 (19.0) | | 46.8 (40.9-52.7) | 38.9 (35.5-42.3) | 45.0 (37.4-53.4) | 43.8 (39.4-48.4) |
|  |  | *P*_trend_ |  |  | | 0.58 | 0.18 | 0.31 | 0.18 |
|  |  | *P* _i_ |  |  | | 0.26 | | 0.81 | |
|  | rs2010750 | GG | 47 (6.5) | 186 (25.7) | | 44.5 (38.8-50.2) | 42.4 (39.5-45.2) | 50.8 (42.9-59.4) | 47.1 (43.2-51.1) |
|  |  | GA | 89 (12.3) | 258 (35.6) | | 43.9 (39.7-48.1) | 42.8 (40.4-45.2) | 46.3 (40.8-52.2) | 47.6 (44.3-51.1) |
|  |  | AA | 37 (5.1) | 107 (14.8) | | 46.6 (40.2-53.1) | 38.9 (35.1-42.7) | 43.9 (35.7-52.9) | 43.7 (38.8-49.0) |
|  |  | *P*_trend_ |  |  | | 0.66 | 0.22 | 0.25 | 0.39 |
|  |  | *P* _i_ |  |  | | 0.33 | | 0.55 | |
| [COMT](../%0DAdditional%20file%201.%20Modifying%20effect%20of%20parity%20on%20the%20association%20between%20SNPs%20and%20mammographic%20density%07%07Gene%07SNP%07%07%07Adjusted%20mean%20mammographic%20densitya%07%07Name%07Reference%20IDb%07Genotype%07N(%25)%07Percent%20density%20(%25)%07Absolute%20density%20(cm2)c%07%07Parity%07%07%07No%07Yes%07No%07Yes%07No%07Yes%07%07ERα%07rs2077647%07TT%0751%20(7.1)%07137%20(19.1)%0744.6%20(39.2-50.1)%0741.8%20(38.4-45.1)%0750.3%20(42.8-58.5)%0746.6%20(42.1-51.4)%07%07%07%07TC%0782%20(11.4)%07270%20(37.6)%0744.6%20(40.3-49.0)%0741.9%20(39.6-44.3)%0746.5%20(40.7-52.7)%0747.4%20(44.2-50.8)%07%07%07%07CC%0739%20(5.4)%07139%20(19.4)%0745.2%20(38.8-51.6)%0742.7%20(39.4-46.0)%0743.4%20(35.4-52.3)%0745.0%20(40.7-49.6)%07%07%07%07Ptrendd%07%07%070.91%070.70%070.24%070.62%07%07%07%07P%20ie%07%07%070.93%070.43%07%07%07rs2234693%07AA%0752%20(7.2)%07157%20(21.7)%0744.0%20(38.5-49.4)%0741.7%20(38.5-44.8)%0750.9%20(43.4-59.1)%0747.6%20(43.3-52.0)%07%07%07%07AG%0787%20(12.0)%07259%20(35.7)%0745.7%20(41.4-49.9)%0741.1%20(38.7-43.5)%0746.6%20(41.0-52.7)%0746.9%20(43.6-50.3)%07%07%07%07GG%0734%20(4.7)%07136%20(18.8)%0744.6%20(37.8-51.4)%0743.3%20(39.9-46.6)%0744.6%20(36.0-54.2)%0744.2%20(39.8-48.8)%07%07%07%07Ptrend%07%07%070.82%070.51%070.28%070.30%07%07%07%07P%20i%07%07%070.91%070.65%07%07%07rs9340799%07AA%0781%20(11.2)%07218%20(30.1)%0744.4%20(40.1-48.8)%0742.2%20(39.6-44.8)%0750.1%20(44.1-56.6)%0749.2%20(45.5-53.0)%07%07%07%07AG%0769%20(9.5)%07252%20(34.8)%0745.0%20(40.2-49.7)%0740.9%20(38.4-43.3)%0743.5%20(37.5-50.1)%0744.9%20(41.7-48.3)%07%07%07%07GG%0723%20(3.2)%0782%20(11.3)%0747.3%20(39.1-55.6)%0744.0%20(39.7-48.3)%0751.3%20(40.1-64.0)%0744.7%20(39.2-50.7)%07%07%07%07Ptrend%07%07%070.58%070.76%070.65%070.11%07%07%07%07P%20i%07%07%070.74%070.70%07%07%07rs2228480%07CC%07126%20(17.4)%07376%20(51.8)%0744.8%20(41.3-48.3)%0741.1%20(39.1-43.1)%0748.1%20(43.4-53.2)%0745.5%20(42.8-48.2)%07%07%07%07CT%0746%20(6.3)%07157%20(21.6)%0745.2%20(39.4-51.0)%0743.0%20(39.9-46.2)%0744.5%20(37.1-52.7)%0748.1%20(43.9-52.6)%07%07%07%07TT%072%20(0.3)%0719%20(2.6)%0716.9%20(-10.7-44.4)%0748.9%20(39.9-57.8)%0729.2%20(8.6-66.9)%0757.3%20(44.4-72.0)%07%07%07%07Ptrend%07%07%070.51%070.09%070.27%070.08%07%07%07%07P%20i%07%07%070.18%070.08%07%07ERβ%07rs3829768%07TT%07173%20(23.9)%07550%20(75.9)%0744.8%20(41.8-47.8)%0741.9%20(40.2-43.5)%0747.2%20(43.1-51.5)%0746.5%20(44.3-48.8)%07%07%07%07TC%071%20(0.1)%071%20(0.1)%07-8.2%20(-47.1-30.7)%0741.6%20(2.4-80.8)%076.0%20(12.3-33.8)%0792.5%20(31.7-187.9)%07%07%07%07Ptrend%07%07%070.008%070.99%070.01%070.17%07%07%07%07P%20i%07%07%070.06%070.006%07%07%07rs1256049%07GG%07162%20(22.3)%07517%20(71.1)%0745.4%20(42.3-48.5)%0741.9%20(40.2-43.6)%0748.0%20(43.7-52.4)%0746.6%20(44.2-49.0)%07%07%07%07GA%0712%20(1.7)%0736%20(5.0)%0737.9%20(26.6-49.2)%0740.8%20(34.3-47.4)%0741.0%20(27.9-56.9)%0746.1%20(37.6-55.6)%07%07%07%07AA%070%070%07%07%07%07%07%07%07%07Ptrend%07%07%070.21%070.76%070.38%070.92%07%07%07%07P%20i%07%07%070.35%070.48%07%07HSD17B1%07rs676387%07GG%0788%20(12.1)%07296%20(40.7)%0746.0%20(41.8-50.2)%0740.4%20(38.2-42.7)%0745.1%20(39.6-51.0)%0745.2%20(42.2-48.4)%07%07%07%07GT%0775%20(10.3)%07212%20(29.2)%0742.8%20(38.3-47.4)%0743.8%20(41.1-46.4)%0748.7%20(42.5-55.2)%0748.4%20(44.7-52.2)%07%07%07%07TT%0711%20(1.5)%0745%20(6.2)%0743.4%20(31.7-55.2)%0742.1%20(36.3-47.9)%0748.8%20(33.8-67.0)%0746.6%20(39.0-55.0)%07%07%07%07Ptrend%07%07%070.37%070.16%070.42%070.34%07%07%07%07P%20i%07%07%070.14%070.80%07%07%07rs598126%07CC%0742%20(5.8)%07149%20(20.6)%0744.5%20(38.4-50.5)%0742.2%20(39.0-45.4)%0751.1%20(42.8-60.3)%0748.1%20(43.8-52.7)%07%07%07%07CT%0787%20(12.1)%07263%20(36.4)%0743.5%20(39.3-47.7)%0743.0%20(40.6-45.4)%0745.9%20(40.4-51.9)%0746.8%20(43.5-50.1)%07%07%07%07TT%0744%20(6.1)%07137%20(19.0)%0746.8%20(40.9-52.7)%0738.9%20(35.5-42.3)%0745.0%20(37.4-53.4)%0743.8%20(39.4-48.4)%07%07%07%07Ptrend%07%07%070.58%070.18%070.31%070.18%07%07%07%07P%20i%07%07%070.26%070.81%07%07%07rs2010750%07GG%0747%20(6.5)%07186%20(25.7)%0744.5%20(38.8-50.2)%0742.4%20(39.5-45.2)%0750.8%20(42.9-59.4)%0747.1%20(43.2-51.1)%07%07%07%07GA%0789%20(12.3)%07258%20(35.6)%0743.9%20(39.7-48.1)%0742.8%20(40.4-45.2)%0746.3%20(40.8-52.2)%0747.6%20(44.3-51.1)%07%07%07%07AA%0737%20(5.1)%07107%20(14.8)%0746.6%20(40.2-53.1)%0738.9%20(35.1-42.7)%0743.9%20(35.7-52.9)%0743.7%20(38.8-49.0)%07%07%07%07Ptrend%07%07%070.66%070.22%070.25%070.39%07%07%07%07P%20i%07%07%070.33%070.55%07%07COMT%07rs4680%07GG%0744%20(6.1)%07143%20(19.7)%0743.1%20(37.1-49.1)%0742.0%20(38.7-45.3)%0743.3%20(35.7-51.7)%0748.8%20(44.3-53.6)%07%07%07%07GA%0796%20(13.2)%07275%20(37.9)%0745.3%20(41.3-49.3)%0743.3%20(41.0-45.7)%0746.4%20(41.1-52.1)%0746.7%20(43.5-50.1)%07%07%07%07AA%0734%20(4.7)%07133%20(18.3)%0743.4%20(36.7-50.0)%0738.6%20(35.2-42.0)%0752.6%20(43.3-62.9)%0743.6%20(39.3-48.2)%07%07%07%07Ptrend%07%07%070.93%070.17%070.15%070.12%07%07%07%07P%20i%07%07%070.47%070.05%07%07CYP1B1%07rs1056836%07CC%0753%20(7.3)%07163%20(22.4)%0746.6%20(41.1-52.1)%0742.8%20(39.7-45.8)%0754.2%20(46.4-62.7)%0747.2%20(43.1-51.5)%07%07%07%07CG%0786%20(11.8)%07282%20(38.8)%0746.3%20(42.0-50.5)%0741.8%20(39.5-44.1)%0747.0%20(41.3-53.0)%0746.4%20(43.3-49.6)%07%07%07%07GG%0735%20(4.8)%07108%20(14.9)%0736.9%20(30.3-43.5)%0740.6%20(36.8-44.3)%0737.0%20(29.5-45.4)%0745.5%20(40.6-50.7)%07%07%07%07Ptrend%07%07%070.04%070.38%070.004%070.62%07%07%07%07P%20i%07%07%070.19%070.02%07%07a%20Analyses%20are%20adjusted%20for%20age%20at%20mammography,%20body%20mass%20index,%20waist-to-hip%20ratio,%20height,%20age%20at%20menarche,%20age%20at%20first%20birth,%20number%20of%20full-term%20pregnancies,%20breastfeeding,%20number%20of%20breast%20biopsies,%20family%20history%20of%20breast%20cancer,%20past%20contraceptive%20and%20hormone%20replacement%20therapy%20uses,%20smoking%20status,%20energy%20and%20alcohol%20intakes,%20physical%20activity%20and%20education%20when%20applicable.%20Except%20for%20modifying%20effect%20of%20parity,%20models%20were%20also%20adjusted%20for%20age%20at%20first%20birth,%20number%20of%20full-term%20pregnancies%20and%20breastfeeding.%0Db%20%20SNPs:%20single%20nucleotide%20polymorphisms.%20They%20are%20identified%20by%20their%20dbSNP%20accession%20number%20at%20http://www.ncbi.nlm.nih.gov/SNP/.%0Dc%20Means%20of%20absolute%20density%20are%20presented%20as%20back-transformed%20values.%0Dd%20%20P%20value%20is%20the%20p%20trend,%20testing%20genotype%20dosage,%20number%20of%20copies%20of%20the%20rare%20allele%20entered%20as%200,%201,%202%20and%20mammographic%20density%20entered%20as%20a%20continuous%20variable.%0De%20%20P%20value%20for%20interaction%20between%20the%20variable%20(parity,%20hormonal%20derivative%20used,%20age%20at%20menarche%20or%20body%20mass%20index)%20and%20the%20genotype%20dosage%20%0Dfrom%20linear%20regression.%07%07) | rs4680 | GG | 44 (6.1) | 143 (19.7) | | 43.1 (37.1-49.1) | 42.0 (38.7-45.3) | 43.3 (35.7-51.7) | 48.8 (44.3-53.6) |
|  |  | GA | 96 (13.2) | 275 (38.0) | | 45.3 (41.3-49.3) | 43.3 (41.0-45.7) | 46.4 (41.1-52.1) | 46.7 (43.5-50.1) |
|  |  | AA | 34 (4.7) | 133 (18.3) | | 43.4 (36.7-50.0) | 38.6 (35.2-42.0) | 52.6 (43.3-62.9) | 43.6 (39.3-48.2) |
|  |  | *P*_trend_ |  |  | | 0.93 | 0.17 | 0.15 | 0.12 |
|  |  | *P* _i_ |  |  | | 0.47 | | 0.05 | |
| [CYP1B1](../%0DAdditional%20file%201.%20Modifying%20effect%20of%20parity%20on%20the%20association%20between%20SNPs%20and%20mammographic%20density%07%07Gene%07SNP%07%07%07Adjusted%20mean%20mammographic%20densitya%07%07Name%07Reference%20IDb%07Genotype%07N(%25)%07Percent%20density%20(%25)%07Absolute%20density%20(cm2)c%07%07Parity%07%07%07No%07Yes%07No%07Yes%07No%07Yes%07%07ERα%07rs2077647%07TT%0751%20(7.1)%07137%20(19.1)%0744.6%20(39.2-50.1)%0741.8%20(38.4-45.1)%0750.3%20(42.8-58.5)%0746.6%20(42.1-51.4)%07%07%07%07TC%0782%20(11.4)%07270%20(37.6)%0744.6%20(40.3-49.0)%0741.9%20(39.6-44.3)%0746.5%20(40.7-52.7)%0747.4%20(44.2-50.8)%07%07%07%07CC%0739%20(5.4)%07139%20(19.4)%0745.2%20(38.8-51.6)%0742.7%20(39.4-46.0)%0743.4%20(35.4-52.3)%0745.0%20(40.7-49.6)%07%07%07%07Ptrendd%07%07%070.91%070.70%070.24%070.62%07%07%07%07P%20ie%07%07%070.93%070.43%07%07%07rs2234693%07AA%0752%20(7.2)%07157%20(21.7)%0744.0%20(38.5-49.4)%0741.7%20(38.5-44.8)%0750.9%20(43.4-59.1)%0747.6%20(43.3-52.0)%07%07%07%07AG%0787%20(12.0)%07259%20(35.7)%0745.7%20(41.4-49.9)%0741.1%20(38.7-43.5)%0746.6%20(41.0-52.7)%0746.9%20(43.6-50.3)%07%07%07%07GG%0734%20(4.7)%07136%20(18.8)%0744.6%20(37.8-51.4)%0743.3%20(39.9-46.6)%0744.6%20(36.0-54.2)%0744.2%20(39.8-48.8)%07%07%07%07Ptrend%07%07%070.82%070.51%070.28%070.30%07%07%07%07P%20i%07%07%070.91%070.65%07%07%07rs9340799%07AA%0781%20(11.2)%07218%20(30.1)%0744.4%20(40.1-48.8)%0742.2%20(39.6-44.8)%0750.1%20(44.1-56.6)%0749.2%20(45.5-53.0)%07%07%07%07AG%0769%20(9.5)%07252%20(34.8)%0745.0%20(40.2-49.7)%0740.9%20(38.4-43.3)%0743.5%20(37.5-50.1)%0744.9%20(41.7-48.3)%07%07%07%07GG%0723%20(3.2)%0782%20(11.3)%0747.3%20(39.1-55.6)%0744.0%20(39.7-48.3)%0751.3%20(40.1-64.0)%0744.7%20(39.2-50.7)%07%07%07%07Ptrend%07%07%070.58%070.76%070.65%070.11%07%07%07%07P%20i%07%07%070.74%070.70%07%07%07rs2228480%07CC%07126%20(17.4)%07376%20(51.8)%0744.8%20(41.3-48.3)%0741.1%20(39.1-43.1)%0748.1%20(43.4-53.2)%0745.5%20(42.8-48.2)%07%07%07%07CT%0746%20(6.3)%07157%20(21.6)%0745.2%20(39.4-51.0)%0743.0%20(39.9-46.2)%0744.5%20(37.1-52.7)%0748.1%20(43.9-52.6)%07%07%07%07TT%072%20(0.3)%0719%20(2.6)%0716.9%20(-10.7-44.4)%0748.9%20(39.9-57.8)%0729.2%20(8.6-66.9)%0757.3%20(44.4-72.0)%07%07%07%07Ptrend%07%07%070.51%070.09%070.27%070.08%07%07%07%07P%20i%07%07%070.18%070.08%07%07ERβ%07rs3829768%07TT%07173%20(23.9)%07550%20(75.9)%0744.8%20(41.8-47.8)%0741.9%20(40.2-43.5)%0747.2%20(43.1-51.5)%0746.5%20(44.3-48.8)%07%07%07%07TC%071%20(0.1)%071%20(0.1)%07-8.2%20(-47.1-30.7)%0741.6%20(2.4-80.8)%076.0%20(12.3-33.8)%0792.5%20(31.7-187.9)%07%07%07%07Ptrend%07%07%070.008%070.99%070.01%070.17%07%07%07%07P%20i%07%07%070.06%070.006%07%07%07rs1256049%07GG%07162%20(22.3)%07517%20(71.1)%0745.4%20(42.3-48.5)%0741.9%20(40.2-43.6)%0748.0%20(43.7-52.4)%0746.6%20(44.2-49.0)%07%07%07%07GA%0712%20(1.7)%0736%20(5.0)%0737.9%20(26.6-49.2)%0740.8%20(34.3-47.4)%0741.0%20(27.9-56.9)%0746.1%20(37.6-55.6)%07%07%07%07AA%070%070%07%07%07%07%07%07%07%07Ptrend%07%07%070.21%070.76%070.38%070.92%07%07%07%07P%20i%07%07%070.35%070.48%07%07HSD17B1%07rs676387%07GG%0788%20(12.1)%07296%20(40.7)%0746.0%20(41.8-50.2)%0740.4%20(38.2-42.7)%0745.1%20(39.6-51.0)%0745.2%20(42.2-48.4)%07%07%07%07GT%0775%20(10.3)%07212%20(29.2)%0742.8%20(38.3-47.4)%0743.8%20(41.1-46.4)%0748.7%20(42.5-55.2)%0748.4%20(44.7-52.2)%07%07%07%07TT%0711%20(1.5)%0745%20(6.2)%0743.4%20(31.7-55.2)%0742.1%20(36.3-47.9)%0748.8%20(33.8-67.0)%0746.6%20(39.0-55.0)%07%07%07%07Ptrend%07%07%070.37%070.16%070.42%070.34%07%07%07%07P%20i%07%07%070.14%070.80%07%07%07rs598126%07CC%0742%20(5.8)%07149%20(20.6)%0744.5%20(38.4-50.5)%0742.2%20(39.0-45.4)%0751.1%20(42.8-60.3)%0748.1%20(43.8-52.7)%07%07%07%07CT%0787%20(12.1)%07263%20(36.4)%0743.5%20(39.3-47.7)%0743.0%20(40.6-45.4)%0745.9%20(40.4-51.9)%0746.8%20(43.5-50.1)%07%07%07%07TT%0744%20(6.1)%07137%20(19.0)%0746.8%20(40.9-52.7)%0738.9%20(35.5-42.3)%0745.0%20(37.4-53.4)%0743.8%20(39.4-48.4)%07%07%07%07Ptrend%07%07%070.58%070.18%070.31%070.18%07%07%07%07P%20i%07%07%070.26%070.81%07%07%07rs2010750%07GG%0747%20(6.5)%07186%20(25.7)%0744.5%20(38.8-50.2)%0742.4%20(39.5-45.2)%0750.8%20(42.9-59.4)%0747.1%20(43.2-51.1)%07%07%07%07GA%0789%20(12.3)%07258%20(35.6)%0743.9%20(39.7-48.1)%0742.8%20(40.4-45.2)%0746.3%20(40.8-52.2)%0747.6%20(44.3-51.1)%07%07%07%07AA%0737%20(5.1)%07107%20(14.8)%0746.6%20(40.2-53.1)%0738.9%20(35.1-42.7)%0743.9%20(35.7-52.9)%0743.7%20(38.8-49.0)%07%07%07%07Ptrend%07%07%070.66%070.22%070.25%070.39%07%07%07%07P%20i%07%07%070.33%070.55%07%07COMT%07rs4680%07GG%0744%20(6.1)%07143%20(19.7)%0743.1%20(37.1-49.1)%0742.0%20(38.7-45.3)%0743.3%20(35.7-51.7)%0748.8%20(44.3-53.6)%07%07%07%07GA%0796%20(13.2)%07275%20(37.9)%0745.3%20(41.3-49.3)%0743.3%20(41.0-45.7)%0746.4%20(41.1-52.1)%0746.7%20(43.5-50.1)%07%07%07%07AA%0734%20(4.7)%07133%20(18.3)%0743.4%20(36.7-50.0)%0738.6%20(35.2-42.0)%0752.6%20(43.3-62.9)%0743.6%20(39.3-48.2)%07%07%07%07Ptrend%07%07%070.93%070.17%070.15%070.12%07%07%07%07P%20i%07%07%070.47%070.05%07%07CYP1B1%07rs1056836%07CC%0753%20(7.3)%07163%20(22.4)%0746.6%20(41.1-52.1)%0742.8%20(39.7-45.8)%0754.2%20(46.4-62.7)%0747.2%20(43.1-51.5)%07%07%07%07CG%0786%20(11.8)%07282%20(38.8)%0746.3%20(42.0-50.5)%0741.8%20(39.5-44.1)%0747.0%20(41.3-53.0)%0746.4%20(43.3-49.6)%07%07%07%07GG%0735%20(4.8)%07108%20(14.9)%0736.9%20(30.3-43.5)%0740.6%20(36.8-44.3)%0737.0%20(29.5-45.4)%0745.5%20(40.6-50.7)%07%07%07%07Ptrend%07%07%070.04%070.38%070.004%070.62%07%07%07%07P%20i%07%07%070.19%070.02%07%07a%20Analyses%20are%20adjusted%20for%20age%20at%20mammography,%20body%20mass%20index,%20waist-to-hip%20ratio,%20height,%20age%20at%20menarche,%20age%20at%20first%20birth,%20number%20of%20full-term%20pregnancies,%20breastfeeding,%20number%20of%20breast%20biopsies,%20family%20history%20of%20breast%20cancer,%20past%20contraceptive%20and%20hormone%20replacement%20therapy%20uses,%20smoking%20status,%20energy%20and%20alcohol%20intakes,%20physical%20activity%20and%20education%20when%20applicable.%20Except%20for%20modifying%20effect%20of%20parity,%20models%20were%20also%20adjusted%20for%20age%20at%20first%20birth,%20number%20of%20full-term%20pregnancies%20and%20breastfeeding.%0Db%20%20SNPs:%20single%20nucleotide%20polymorphisms.%20They%20are%20identified%20by%20their%20dbSNP%20accession%20number%20at%20http://www.ncbi.nlm.nih.gov/SNP/.%0Dc%20Means%20of%20absolute%20density%20are%20presented%20as%20back-transformed%20values.%0Dd%20%20P%20value%20is%20the%20p%20trend,%20testing%20genotype%20dosage,%20number%20of%20copies%20of%20the%20rare%20allele%20entered%20as%200,%201,%202%20and%20mammographic%20density%20entered%20as%20a%20continuous%20variable.%0De%20%20P%20value%20for%20interaction%20between%20the%20variable%20(parity,%20hormonal%20derivative%20used,%20age%20at%20menarche%20or%20body%20mass%20index)%20and%20the%20genotype%20dosage%20%0Dfrom%20linear%20regression.%07%07) | rs1056836 | CC | 53 (7.3) | 163 (22.4) | | 46.6 (41.1-52.1) | 42.8 (39.7-45.8) | 54.2 (46.4-62.7) | 47.2 (43.1-51.5) |
|  |  | CG | 86 (11.8) | 282 (38.8) | | 46.3 (42.0-50.5) | 41.8 (39.5-44.1) | 47.0 (41.3-53.0) | 46.4 (43.3-49.6) |
|  |  | GG | 35 (4.8) | 108 (14.8) | | 36.9 (30.3-43.5) | 40.6 (36.8-44.3) | 37.0 (29.5-45.4) | 45.5 (40.6-50.7) |
|  |  | *P*_trend_ |  |  | | 0.04 | 0.38 | 0.004 | 0.62 |
|  |  | *P* _i_ |  |  | | 0.19 | | 0.02 | |
| [^a^](../%0DAdditional%20file%201.%20Modifying%20effect%20of%20parity%20on%20the%20association%20between%20SNPs%20and%20mammographic%20density%07%07Gene%07SNP%07%07%07Adjusted%20mean%20mammographic%20densitya%07%07Name%07Reference%20IDb%07Genotype%07N(%25)%07Percent%20density%20(%25)%07Absolute%20density%20(cm2)c%07%07Parity%07%07%07No%07Yes%07No%07Yes%07No%07Yes%07%07ERα%07rs2077647%07TT%0751%20(7.1)%07137%20(19.1)%0744.6%20(39.2-50.1)%0741.8%20(38.4-45.1)%0750.3%20(42.8-58.5)%0746.6%20(42.1-51.4)%07%07%07%07TC%0782%20(11.4)%07270%20(37.6)%0744.6%20(40.3-49.0)%0741.9%20(39.6-44.3)%0746.5%20(40.7-52.7)%0747.4%20(44.2-50.8)%07%07%07%07CC%0739%20(5.4)%07139%20(19.4)%0745.2%20(38.8-51.6)%0742.7%20(39.4-46.0)%0743.4%20(35.4-52.3)%0745.0%20(40.7-49.6)%07%07%07%07Ptrendd%07%07%070.91%070.70%070.24%070.62%07%07%07%07P%20ie%07%07%070.93%070.43%07%07%07rs2234693%07AA%0752%20(7.2)%07157%20(21.7)%0744.0%20(38.5-49.4)%0741.7%20(38.5-44.8)%0750.9%20(43.4-59.1)%0747.6%20(43.3-52.0)%07%07%07%07AG%0787%20(12.0)%07259%20(35.7)%0745.7%20(41.4-49.9)%0741.1%20(38.7-43.5)%0746.6%20(41.0-52.7)%0746.9%20(43.6-50.3)%07%07%07%07GG%0734%20(4.7)%07136%20(18.8)%0744.6%20(37.8-51.4)%0743.3%20(39.9-46.6)%0744.6%20(36.0-54.2)%0744.2%20(39.8-48.8)%07%07%07%07Ptrend%07%07%070.82%070.51%070.28%070.30%07%07%07%07P%20i%07%07%070.91%070.65%07%07%07rs9340799%07AA%0781%20(11.2)%07218%20(30.1)%0744.4%20(40.1-48.8)%0742.2%20(39.6-44.8)%0750.1%20(44.1-56.6)%0749.2%20(45.5-53.0)%07%07%07%07AG%0769%20(9.5)%07252%20(34.8)%0745.0%20(40.2-49.7)%0740.9%20(38.4-43.3)%0743.5%20(37.5-50.1)%0744.9%20(41.7-48.3)%07%07%07%07GG%0723%20(3.2)%0782%20(11.3)%0747.3%20(39.1-55.6)%0744.0%20(39.7-48.3)%0751.3%20(40.1-64.0)%0744.7%20(39.2-50.7)%07%07%07%07Ptrend%07%07%070.58%070.76%070.65%070.11%07%07%07%07P%20i%07%07%070.74%070.70%07%07%07rs2228480%07CC%07126%20(17.4)%07376%20(51.8)%0744.8%20(41.3-48.3)%0741.1%20(39.1-43.1)%0748.1%20(43.4-53.2)%0745.5%20(42.8-48.2)%07%07%07%07CT%0746%20(6.3)%07157%20(21.6)%0745.2%20(39.4-51.0)%0743.0%20(39.9-46.2)%0744.5%20(37.1-52.7)%0748.1%20(43.9-52.6)%07%07%07%07TT%072%20(0.3)%0719%20(2.6)%0716.9%20(-10.7-44.4)%0748.9%20(39.9-57.8)%0729.2%20(8.6-66.9)%0757.3%20(44.4-72.0)%07%07%07%07Ptrend%07%07%070.51%070.09%070.27%070.08%07%07%07%07P%20i%07%07%070.18%070.08%07%07ERβ%07rs3829768%07TT%07173%20(23.9)%07550%20(75.9)%0744.8%20(41.8-47.8)%0741.9%20(40.2-43.5)%0747.2%20(43.1-51.5)%0746.5%20(44.3-48.8)%07%07%07%07TC%071%20(0.1)%071%20(0.1)%07-8.2%20(-47.1-30.7)%0741.6%20(2.4-80.8)%076.0%20(12.3-33.8)%0792.5%20(31.7-187.9)%07%07%07%07Ptrend%07%07%070.008%070.99%070.01%070.17%07%07%07%07P%20i%07%07%070.06%070.006%07%07%07rs1256049%07GG%07162%20(22.3)%07517%20(71.1)%0745.4%20(42.3-48.5)%0741.9%20(40.2-43.6)%0748.0%20(43.7-52.4)%0746.6%20(44.2-49.0)%07%07%07%07GA%0712%20(1.7)%0736%20(5.0)%0737.9%20(26.6-49.2)%0740.8%20(34.3-47.4)%0741.0%20(27.9-56.9)%0746.1%20(37.6-55.6)%07%07%07%07AA%070%070%07%07%07%07%07%07%07%07Ptrend%07%07%070.21%070.76%070.38%070.92%07%07%07%07P%20i%07%07%070.35%070.48%07%07HSD17B1%07rs676387%07GG%0788%20(12.1)%07296%20(40.7)%0746.0%20(41.8-50.2)%0740.4%20(38.2-42.7)%0745.1%20(39.6-51.0)%0745.2%20(42.2-48.4)%07%07%07%07GT%0775%20(10.3)%07212%20(29.2)%0742.8%20(38.3-47.4)%0743.8%20(41.1-46.4)%0748.7%20(42.5-55.2)%0748.4%20(44.7-52.2)%07%07%07%07TT%0711%20(1.5)%0745%20(6.2)%0743.4%20(31.7-55.2)%0742.1%20(36.3-47.9)%0748.8%20(33.8-67.0)%0746.6%20(39.0-55.0)%07%07%07%07Ptrend%07%07%070.37%070.16%070.42%070.34%07%07%07%07P%20i%07%07%070.14%070.80%07%07%07rs598126%07CC%0742%20(5.8)%07149%20(20.6)%0744.5%20(38.4-50.5)%0742.2%20(39.0-45.4)%0751.1%20(42.8-60.3)%0748.1%20(43.8-52.7)%07%07%07%07CT%0787%20(12.1)%07263%20(36.4)%0743.5%20(39.3-47.7)%0743.0%20(40.6-45.4)%0745.9%20(40.4-51.9)%0746.8%20(43.5-50.1)%07%07%07%07TT%0744%20(6.1)%07137%20(19.0)%0746.8%20(40.9-52.7)%0738.9%20(35.5-42.3)%0745.0%20(37.4-53.4)%0743.8%20(39.4-48.4)%07%07%07%07Ptrend%07%07%070.58%070.18%070.31%070.18%07%07%07%07P%20i%07%07%070.26%070.81%07%07%07rs2010750%07GG%0747%20(6.5)%07186%20(25.7)%0744.5%20(38.8-50.2)%0742.4%20(39.5-45.2)%0750.8%20(42.9-59.4)%0747.1%20(43.2-51.1)%07%07%07%07GA%0789%20(12.3)%07258%20(35.6)%0743.9%20(39.7-48.1)%0742.8%20(40.4-45.2)%0746.3%20(40.8-52.2)%0747.6%20(44.3-51.1)%07%07%07%07AA%0737%20(5.1)%07107%20(14.8)%0746.6%20(40.2-53.1)%0738.9%20(35.1-42.7)%0743.9%20(35.7-52.9)%0743.7%20(38.8-49.0)%07%07%07%07Ptrend%07%07%070.66%070.22%070.25%070.39%07%07%07%07P%20i%07%07%070.33%070.55%07%07COMT%07rs4680%07GG%0744%20(6.1)%07143%20(19.7)%0743.1%20(37.1-49.1)%0742.0%20(38.7-45.3)%0743.3%20(35.7-51.7)%0748.8%20(44.3-53.6)%07%07%07%07GA%0796%20(13.2)%07275%20(37.9)%0745.3%20(41.3-49.3)%0743.3%20(41.0-45.7)%0746.4%20(41.1-52.1)%0746.7%20(43.5-50.1)%07%07%07%07AA%0734%20(4.7)%07133%20(18.3)%0743.4%20(36.7-50.0)%0738.6%20(35.2-42.0)%0752.6%20(43.3-62.9)%0743.6%20(39.3-48.2)%07%07%07%07Ptrend%07%07%070.93%070.17%070.15%070.12%07%07%07%07P%20i%07%07%070.47%070.05%07%07CYP1B1%07rs1056836%07CC%0753%20(7.3)%07163%20(22.4)%0746.6%20(41.1-52.1)%0742.8%20(39.7-45.8)%0754.2%20(46.4-62.7)%0747.2%20(43.1-51.5)%07%07%07%07CG%0786%20(11.8)%07282%20(38.8)%0746.3%20(42.0-50.5)%0741.8%20(39.5-44.1)%0747.0%20(41.3-53.0)%0746.4%20(43.3-49.6)%07%07%07%07GG%0735%20(4.8)%07108%20(14.9)%0736.9%20(30.3-43.5)%0740.6%20(36.8-44.3)%0737.0%20(29.5-45.4)%0745.5%20(40.6-50.7)%07%07%07%07Ptrend%07%07%070.04%070.38%070.004%070.62%07%07%07%07P%20i%07%07%070.19%070.02%07%07a%20Analyses%20are%20adjusted%20for%20age%20at%20mammography,%20body%20mass%20index,%20waist-to-hip%20ratio,%20height,%20age%20at%20menarche,%20age%20at%20first%20birth,%20number%20of%20full-term%20pregnancies,%20breastfeeding,%20number%20of%20breast%20biopsies,%20family%20history%20of%20breast%20cancer,%20past%20contraceptive%20and%20hormone%20replacement%20therapy%20uses,%20smoking%20status,%20energy%20and%20alcohol%20intakes,%20physical%20activity%20and%20education%20when%20applicable.%20Except%20for%20modifying%20effect%20of%20parity,%20models%20were%20also%20adjusted%20for%20age%20at%20first%20birth,%20number%20of%20full-term%20pregnancies%20and%20breastfeeding.%0Db%20%20SNPs:%20single%20nucleotide%20polymorphisms.%20They%20are%20identified%20by%20their%20dbSNP%20accession%20number%20at%20http://www.ncbi.nlm.nih.gov/SNP/.%0Dc%20Means%20of%20absolute%20density%20are%20presented%20as%20back-transformed%20values.%0Dd%20%20P%20value%20is%20the%20p%20trend,%20testing%20genotype%20dosage,%20number%20of%20copies%20of%20the%20rare%20allele%20entered%20as%200,%201,%202%20and%20mammographic%20density%20entered%20as%20a%20continuous%20variable.%0De%20%20P%20value%20for%20interaction%20between%20the%20variable%20(parity,%20hormonal%20derivative%20used,%20age%20at%20menarche%20or%20body%20mass%20index)%20and%20the%20genotype%20dosage%20%0Dfrom%20linear%20regression.%07%07) Analyses are adjusted for age at mammography, body mass index, waist-to-hip ratio, height, age at menarche, number of breast biopsies, family history of breast cancer, past contraceptive and hormone replacement therapy uses, smoking status, energy and alcohol intakes, physical activity and education when applicable. Except for modifying effect of parity, models were also adjusted for age at first birth, number of full-term pregnancies and breastfeeding. CI: confidence interval.  ^b^ SNPs: single nucleotide polymorphisms. They are identified by their dbSNP accession number at http://www.ncbi.nlm.nih.gov/SNP/.  ^c^ Means of absolute density are presented as back-transformed values.  ^d^ *P* value is the *P* trend, testing genotype dosage, number of copies of the rare allele entered as 0, 1, 2 and mammographic density entered as a continuous variable.  ^e^ *P* value for interaction between the variable (parity, hormonal derivative used, age at menarche or body mass index) and the genotype dosage from linear regression. ^f^ N/A: not applicable. | | | | | | | | | |
