# Supplementary material for: Polymorphisms in genes involved in the estrogen pathway and mammographic density
Source: BMC Cancer. 2010 Nov 22;10:636. doi: 10.1186/1471-2407-10-636 (PMC3000407; doi:10.1186/1471-2407-10-636)
Supplement: Additional file 2 — Modifying effect of hormonal derivatives used on the association between SNPs and mammographic density. [file 1471-2407-10-636-S2.DOC]

| **Additional file 2. Modifying effect of hormonal derivatives used on the association between SNPs and mammographic density** | | | | | | | | | |
| --- | --- | --- | --- | --- | --- | --- | --- | --- | --- |
| Gene | SNP |  |  | | | Adjusted mean mammographic density (95% CI)^a^ | | | |
| name | reference ID^b^ | Genotype | N(%) | | | Percent density (%) | | Absolute density (cm^2^)^c^ | |
| **Hormonal derivatives used :** | | | **No** | | **Yes** | **No** | **Yes** | **No** | **Yes** |
| ERα | rs2077647 | TT | 14 (2.0) | 174 (24.2) | | 42.8 (32.2-53.5) | 42.6 (39.6-45.6) | 45.1 (32.1-60.7) | 47.9 (43.9-52.2) |
|  |  | TC | 29 (4.0) | 323 (45.0) | | 42.8 (35.5-50.2) | 42.5 (40.3-44.6) | 42.5 (33.5-52.7) | 47.4 (44.5-50.5) |
|  |  | CC | 12 (1.7) | 166 (23.1) | | 53.0 (41.6-64.3) | 42.6 (39.6-45.6) | 61.2 (44.6-80.7) | 43.6 (39.7-47.7) |
|  |  | *P*_trend_^d^ |  |  | | 0.22 | 0.99 | 0.19 | 0.14 |
|  |  | *P* _i_^e^ |  |  | | 0.24 | | 0.10 | |
|  | rs2234693 | AA | 16 (2.2) | 193 (26.6) | | 42.3 (32.3-52.2) | 42.3 (39.5-45.2) | 44.5 (32.4-58.9) | 48.7 (44.9-52.8) |
|  |  | AG | 25 (3.5) | 321 (44.3) | | 43.8 (35.9-51.7) | 41.9 (39.8-44.1) | 44.0 (34.2-55.1) | 46.9 (43.9-49.9) |
|  |  | GG | 13 (1.8) | 157 (21.7) | | 52.1 (41.2-63.0) | 43.1 (39.9-46.2) | 57.4 (42.0-75.3) | 43.4 (39.4-47.6) |
|  |  | *P*_trend_ |  |  | | 0.20 | 0.77 | 0.25 | 0.07 |
|  |  | *P* _i_ |  |  | | 0.25 | | 0.11 | |
|  | rs9340799 | AA | 23 (3.2) | 276 (38.1) | | 42.3 (33.9-50.8) | 42.7 (40.4-45.1) | 45.2 (34.6-57.4) | 49.7 (46.4-53.1) |
|  |  | AG | 22 (3.0) | 299 (41.2) | | 45.6 (37.2-54.0) | 41.5 (39.2-43.7) | 44.3 (34.0-56.2) | 44.6 (41.6-47.6) |
|  |  | GG | 9 (1.2) | 96 (13.2) | | 53.1 (40.1-66.1) | 44.2 (40.2-48.2) | 61.3 (42.5-83.9) | 45.1 (39.9-50.5) |
|  |  | *P*_trend_ |  |  | | 0.19 | 0.84 | 0.25 | 0.05 |
|  |  | *P* _i_ |  |  | | 0.23 | | 0.10 | |
|  | rs2228480 | CC | 39 (5.4) | 463 (63.8) | | 45.8 (39.4-52.3) | 41.7 (39.9-43.5) | 48.5 (39.9-58.0) | 45.9 (43.5-48.4) |
|  |  | CT | 15 (2.1) | 188 (25.9) | | 43.4 (33.3-53.6) | 43.5 (40.7-46.4) | 43.4 (31.1-57.9) | 47.4 (43.5-51.4) |
|  |  | TT | 1 (0.1) | 20 (2.8) | | 40.8 (1.4-80.2) | 47.2 (38.4-56.0) | 41.9 (8.2-110.2) | 56.9 (44.3-71.2) |
|  |  | *P*_trend_ |  |  | | 0.65 | 0.13 | 0.52 | 0.16 |
|  |  | *P* _i_ |  |  | | 0.41 | | 0.32 | |
| ERβ | rs3829768 | TT | 55 (7.6) | 668 (92.1) | | N/A^f^ | N/A | N/A | N/A |
|  |  | TC | 0 (0) | 2 (0.3) | | N/A | N/A | N/A | N/A |
|  |  | *P*_trend_ |  |  | | N/A | N/A | N/A | N/A |
|  |  | *P* _i_ |  |  | | N/A | | N/A | |
|  | rs1256049 | GG | 50 (6.9) | 629 (86.5) | | 45.5 (39.9-51.2) | 42.5 (41.0-44.1) | 48.7 (41.2-57.0) | 46.7 (44.6-48.9) |
|  |  | GA | 4 (0.6) | 44 (6.1) | | 45.9 (26.0-65.9) | 39.4 (33.5-45.3) | 31.2 (13.8-57.3) | 45.9 (38.2-54.3) |
|  |  | *P*_trend_ |  |  | | 0.97 | 0.32 | 0.18 | 0.84 |
|  |  | *P* _i_ |  |  | | 0.74 | | 0.22 | |
| HSD17B1 | rs676387 | GG | 32 (4.4) | 352 (48.4) | | 47.3 (40.3-54.3) | 41.3 (39.2-43.4) | 49.5 (40.1-59.9) | 44.8 (42.1-47.6) |
|  |  | GT | 20 (2.8) | 267 (36.7) | | 42.5 (33.5-51.4) | 43.4 (41.0-45.8) | 45.2 (34.0-58.2) | 48.5 (45.3-51.9) |
|  |  | TT | 3 (0.4) | 53 (7.3) | | 38.6 (15.9-61.3) | 42.9 (37.6-48.3) | 32.8 (13.0-64.1) | 48.2 (41.0-56.0) |
|  |  | *P*_trend_ |  |  | | 0.31 | 0.25 | 0.30 | 0.11 |
|  |  | *P* _i_ |  |  | | 0.20 | | 0.15 | |
|  | rs598126 | CC | 14 (1.9) | 177 (24.5) | | 38.3 (27.7-49.0) | 43.0 (40.1-45.9) | 38.1 (26.3-52.4) | 49.3 (45.2-53.5) |
|  |  | CT | 24 (3.3) | 326 (45.2) | | 44.2 (36.2-52.2) | 43.0 (40.9-45.2) | 45.4 (35.2-56.9) | 46.6 (43.7-49.7) |
|  |  | TT | 16 (2.2) | 165 (22.9) | | 52.0 (42.1-61.8) | 39.9 (36.9-42.9) | 56.6 (42.6-72.7) | 43.1 (39.2-47.2) |
|  |  | *P*_trend_ |  |  | | 0.06 | 0.16 | 0.06 | 0.04 |
|  |  | *P* _i_ |  |  | | 0.03 | | 0.02 | |
|  | rs2010750 | GG | 15 (2.1) | 218 (30.1) | | 38.2 (27.9-48.5) | 43.1 (40.5-45.8) | 37.9 (26.5-51.7) | 48.2 (44.6-52.0) |
|  |  | GA | 25 (3.5) | 322 (44.5) | | 44.7 (36.8-52.5) | 42.9 (40.8-45.1) | 45.8 (35.8-57.2) | 47.4 (44.4-50.4) |
|  |  | AA | 15 (2.1) | 129 (17.8) | | 52.9 (42.8-63.0) | 39.6 (36.2-43.0) | 58.7 (44.1-75.7) | 42.4 (38.0-47.0) |
|  |  | *P*_trend_ |  |  | | 0.04 | 0.16 | 0.04 | 0.08 |
|  |  | *P* _i_ |  |  | | 0.02 | | 0.01 | |
| COMT | rs4680 | GG | 15 (2.1) | 172 (23.7) | | 47.5 (37.3-57.6) | 41.8 (38.9-44.8) | 55.0 (40.9-71.4) | 47.0 (43.0-51.2) |
|  |  | GA | 29 (4.0) | 342 (47.2) | | 49.1 (41.7-56.6) | 43.3 (41.2-45.4) | 50.2 (40.2-61.5) | 46.1 (43.3-49.0) |
|  |  | AA | 11 (1.5) | 156 (21.5) | | 31.8 (20.1-43.6) | 40.2 (37.1-43.3) | 30.6 (19.4-44.9) | 46.6 (42.5-51.0) |
|  |  | *P*_trend_ |  |  | | 0.07 | 0.50 | 0.02 | 0.93 |
|  |  | *P* _i_ |  |  | | 0.12 | | 0.03 | |
| CYP1B1 | rs1056836 | CC | 14 (1.9) | 202 (27.8) | | 41.1 (30.7-51.5) | 43.9 (41.1-46.6) | 41.9 (29.6-56.6) | 49.3 (45.5-53.3) |
|  |  | CG | 31 (4.3) | 337 (46.4) | | 46.5 (39.4-53.5) | 42.5 (40.3-44.6) | 48.2 (38.8-58.6) | 46.3 (43.4-49.2) |
|  |  | GG | 10 (1.4) | 133 (18.3) | | 46.7 (34.1-59.2) | 39.3 (35.9-42.7) | 50.9 (34.6-70.7) | 43.1 (38.8-47.7) |
|  |  | *P*_trend_ |  |  | | 0.46 | 0.05 | 0.40 | 0.04 |
|  |  | *P* _i_ |  |  | | 0.22 | | 0.18 | |
| ^a^ Analyses are adjusted for age at mammography, body mass index, waist-to-hip ratio, height, age at menarche, number of breast biopsies, family history of breast cancer, past contraceptive and hormone replacement therapy uses, smoking status, energy and alcohol intakes, physical activity and education when applicable. Except for modifying effect of parity, models were also adjusted for age at first birth, number of full-term pregnancies and breastfeeding. CI: confidence interval.  ^b^ SNPs: single nucleotide polymorphisms. They are identified by their dbSNP accession number at http://www.ncbi.nlm.nih.gov/SNP/.  ^c^ Means of absolute density are presented as back-transformed values.  ^d^ *P* value is the *P* trend, testing genotype dosage, number of copies of the rare allele entered as 0, 1, 2 and mammographic density entered as a continuous variable.  ^e^ *P* value for interaction between the variable (parity, hormonal derivative used, age at menarche or body mass index) and the genotype dosage from linear regression. ^f^ N/A: not applicable. | | | | | | | | | |
